# How does academia respond to the burden of infectious and parasitic disease?

**DOI:** 10.1186/s12961-022-00889-0

**Published:** 2022-08-13

**Authors:** Wenjing Zhao, Lili Wang, Lin Zhang

**Affiliations:** 1 School of Information Management, Wuhan University, Wuhan, China; 2 Center for Science, Technology & Education Assessment (CSTEA), Wuhan University, Wuhan, China; 3 UNU-MERIT, Maastricht University, Maastricht, The Netherlands; 4 Centre for R&D Monitoring (ECOOM) and Department of MSI, KU Leuven, Leuven, Belgium

**Keywords:** Infectious and parasitic diseases (IPDs), Academic research, Response patterns, Disease burden, DALYs

## Abstract

**Background:**

Academic research is one of the main avenues through which humans can fight the threat of infectious diseases. However, there have been concerns regarding whether the academic system has provided sufficient efforts to fight infectious diseases we potentially face. Answering these questions could contribute to evidence-based recommendations for setting research priorities and third-mission policies.

**Methods:**

With a focus on one of the most common categories of communicable diseases, infectious and parasitic diseases (IPDs), we searched Web of Science for articles and reviews relevant to IPDs published during the period 2000–2019 and retrieved WHO data on disease burden in corresponding years. The academic response patterns were explored by IPD subcategory and by human development level (an index established by the United Nations). We conduct the analysis in particular to gain insight into the dynamic relationship between disease burden and research effort on IPDs, scientific efforts contributed by countries with different development levels, and the variation trends in international joint efforts.

**Results:**

The greatest burden of IPDs is clustered in the developing regions of Africa, but has received academic response from both developed and developing countries. Highly developed countries dominate the ranks of academic research in this area, yet there is also a clear increase in research efforts from the countries most affected, despite their low human development scale. In fact, the overall analysis reveals an improved capability for addressing local problems from African regions. In terms of international collaboration, highly developed countries such as the United States and United Kingdom have commonly collaborated with needy regions, whereas prolific but developing nations, like China, have not.

**Conclusions:**

From a global perspective, academia has positively responded to health needs caused by IPDs. Although the relevant research output contribution is primarily from the highly developed countries, concentrated and specialized efforts from the undeveloped regions to ease their local burden can be clearly observed. Our findings also indicate a tendency to focus more on local health needs for both developed and undeveloped regions. The insights revealed in this study should benefit a more informed and systemic plan of research priorities.

## Background

Infectious diseases have for centuries been one of the leading causes of death and disability, and despite all human endeavours, they still present a growing challenge to health and social progress [1, 2]. Most recently, the COVID-19 pandemic has shaken up global structures in a range of ways, leaving in its wake an urgent health need that has required rapid and innovative research to control. Biomedical research, with its principles of better patient treatment and illness prevention [3], has been regarded as a core approach to fighting not just COVID-19 but any infectious disease that would threaten our safety. And what the COVID-19 crisis has shown us is that academia can, and will, “turn on a dime” to respond to emergent health needs. As evidence of academia’s adroit response, Zhang et al. [4] point to marked increases in the number of publications, while both Fry et al. [5] and Lee and Haupt [6] acknowledge changing patterns in international collaborations. However, beyond these observations of academia’s tremendous response to COVID-19 and similar public health emergencies of international concern (PHEICs) as defined by WHO, patterns of research into disease burden caused by commonly existing and long-standing infectious disease is an area that warrants further research. Such research could contribute to evidence-based recommendations for setting research priorities or third-mission^1^ policies that help to mitigate some of the negative impacts of infectious diseases.

Long-standing infectious diseases are those that have existed for long periods in human history rather than emerging as outbreaks, epidemics or pandemics. They span many different conditions and presentations, from HIV to nematode infestations to hepatitis and leprosy. We examine 11 major subcategories of infectious and parasitic diseases in this study, collectively referring to them as IPDs. Although the burden of IPDs has decreased over the past few decades [2], the mortality rate has remained intolerably high. According to WHO [7], IPDs were responsible for the deaths of more than 5 million people in 2019—mostly in low- and middle-income countries. With the potential to improve the quality of life by identifying treatment regimens, biomedical research can play a vital role in combatting infectious diseases that affect impoverished populations [8]. Efficient and sufficient efforts from the academic community are required to respond to such health challenges. Indeed, a comprehensive understanding of the features of an academic response to health issues is extremely difficult to achieve, because research interests and priorities are shaped by many interacting social, economic and political factors. However, a systematic study of the dynamic relationship between disease burden and research effort on IPDs, the scientific contributions of countries with different development levels, and the variation trends in international joint efforts is an insightful exercise and useful source for expeditious and efficient strategic research plans. In this study, we adopt the Human Development Index (HDI) developed by the United Nations Development Programme (UNDP) [9] to classify human development levels into four groups. This index consists of factors such as life expectancy, education and per capita income and provides insight into the potential that could be achieved by a country if there were no inequality [10].

The extent to which researchers have responded to the health needs caused by infectious diseases reveals the connections between the so-called supply side of science and knowledge and the demand side in health fields [11]. Academic research is a complex, collaborative and goal-oriented activity [12], driven by diverse individual and social factors [13]. At the individual end of the spectrum, we have, for example, the curiosity of the researcher [14]. At the societal end, we have pressure to solve the particular problems society deems important [15]. However, what needs to be emphasized is that science itself is not a closed and autarkic system. Its development relies greatly on the provision of resources from other parts of society. As such, academic research is naturally expected to produce knowledge that addresses particular needs of society [16]. The huge impetus and benefits to social development that science brings are being increasingly recognized. Accordingly, science policy is gradually bending towards finding answers for both pressing local issues and the world’s grand challenges [17]. Consequently, the relationship between science and society is changing [16] from one where “mad geniuses” are largely left to pursue their own interests, to another where “educated technicians” are increasingly being asked to make substantial and specific contributions to address society’s needs. In other words, although the trajectory of science and technology is influenced by various factors [15, 18, 19], a basic consensus has been reached where research priorities are, to a certain extent, set according to the needs of society [20–22].

Indeed, assessing the research effort required to address complex global challenges has been drawing increasing attention from scholars in many fields—environmental and energy science [23], agriculture [15] and health [24–26], to name a few. In disciplines related to health, such as biomedicine, there is an especially high expectation to focus on the diseases causing the greatest burden [17, 27]. Appropriate research outcomes include improving healthcare capabilities and providing valuable information on disease trends, insights into patterns of care, effective treatments, of course, and so on [28]. Even though each type of such research contribution is affected by a vast range of factors, exploring the long-term correlations between the research effort and disease burden is a first step in broadening our understanding of this interplay, which can lead to a more informed and systemic plan of research priorities.

As metrics that reflect research effort, prior studies have considered academic research output [29, 30], funding [17, 21, 31] and clinical trials [32, 33]. The most common among these is research output, which is what we have used for this study. Because of the heterogeneous nature, it is important to focus on particular regions and/or diseases, such as medical research in Africa [30], India [29] or Palestine [34], or specific chronic respiratory diseases in Europe [35], diabetes in five small countries in Europe [36], neglected tropical diseases (NTDs) in Brazil [37], or cancer research in 29 countries [24] or only in China [25]. At the global level, Yegros-Yegros et al. [38] used research output indexed by MEDLINE and WHO data from the Global Burden of Disease 2010 report to explore the relationships between research effort and health needs for 134 diseases. The authors concluded that research output is heavily concentrated in high-income countries and is mainly focused on the countries’ own health needs. As a result, there is a relative lack of attention to diseases in lower-income countries. Similar results are reported from analyses conducted by Hagenaars et al. [27]. Here, the data indicated an uneven distribution of scientific advances across societal sectors and their diverse demands.

It is well acknowledged that health research resources are unequally distributed. Evans et al. [26] found that many of the health needs associated with infectious diseases that predominantly affect poor populations do not attract attention from the researchers in developed countries. This paper raises the concern that, in general, researchers are not devoting relatively equal effort to the needs of the “poor populations deficient in their own research infrastructure” [26]. In line with this, other studies also point out the need for a better fit between research effort and global burden [39–41]. These arguments typically rest on the long tradition of medical research—to reduce health inequality and ultimately realize universal well-being [26]. Like Evans and colleagues, these scholars argue that those rich countries should invest in research and development (R&D) strategies that address the specific health problems of poor populations, due to the vast discrepancy between disease burden and research capacity in low- to middle-income countries [40, 41]. In contrast, another group of scholars insist that local researchers should address local problems [21, 42], and emphasize the need to justify public spending on health research to taxpayers [43]. Yet, despite all this debate, few studies have comprehensively examined where and how the academic community exerts their research efforts in terms of disease burden on a global scale. In particular, no study has done so with IPDs, which tend to have a low disease burden in high-income countries and a very high disease burden in the lowest-income countries.

The other dimension through which we analyse response patterns is academic collaboration. This element of research is of great significance in that it presents how scholars from different countries with varying backgrounds jointly defend against diseases. Several studies have investigated the features of collaborative medical research between countries. For instance, Kozma and Calero-Medina [44] examined the role of South African scholars in intercontinental scientific collaborations in tropical medicine, immunology and other relevant fields, while Zacca-González et al. [45] looked at international collaborations and productivity in Latin America. However, few studies have integrated information on disease burden into their analysis. Thus, questions over whether countries collaborate to address disease burden deserve investigation—especially with respect to high-burden diseases in underdeveloped regions.

In short, this research gives rise to the following research questions:

1. What is the dynamic relationship between research effort and the burden of IPDs?
2. What roles do countries at various levels of human development play in contributing to scientific research related to IPDs?
3. How have scholars from countries at various levels of human development jointly defended against IPDs?

The rest of this paper proceeds as follows. The next section presents the data, methods and tools used in this analysis, including a primer on IPDs classifications and the sources of data used to explore IPDs research. The main results and our analysis are provided in the third section. The last section contains the conclusion and discussion of results, including the limitations and reflections on future work.

## Methods

**Fig. 1:**
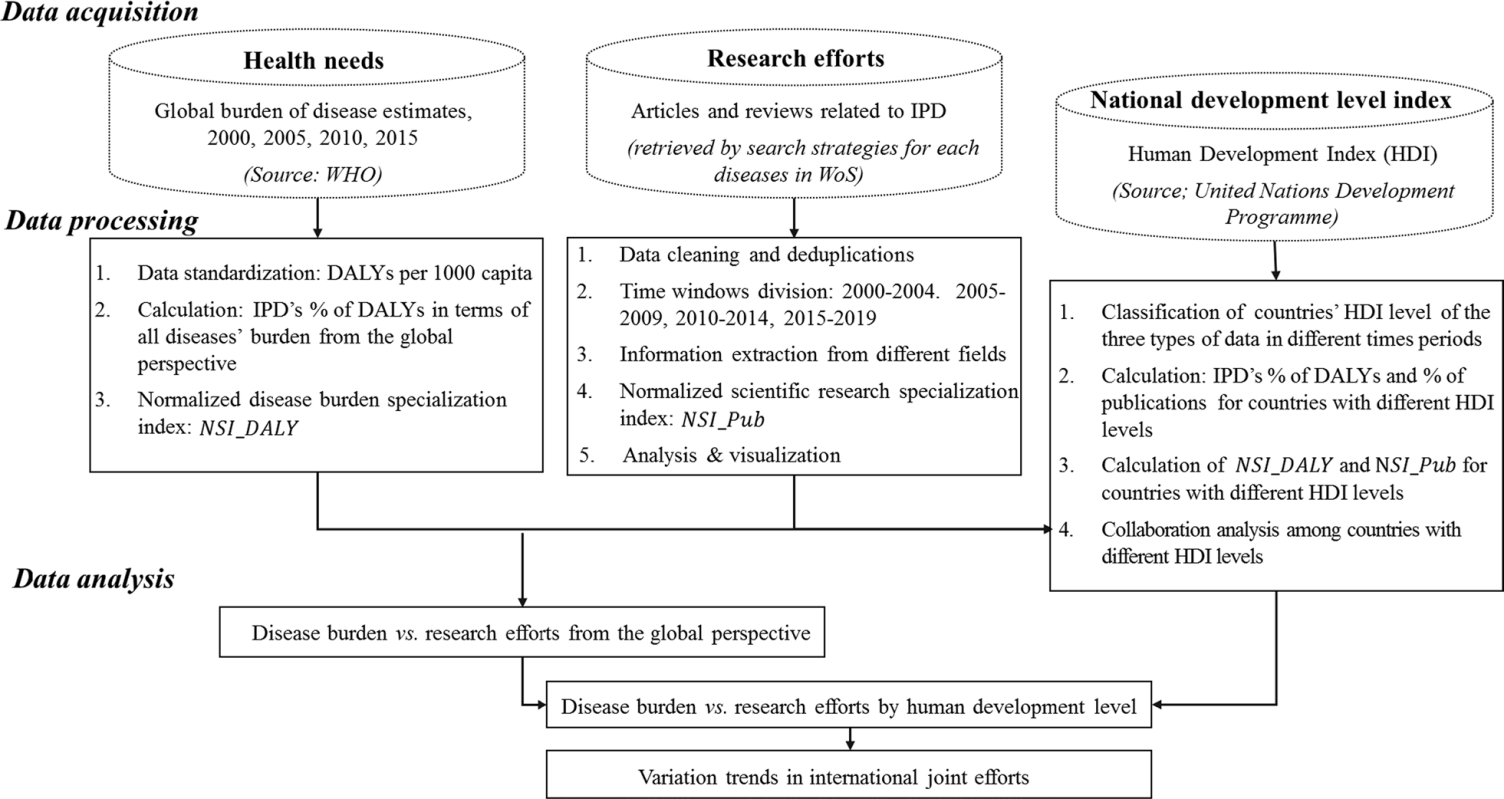
Research framework

Figure 1 depicts the framework used to conduct this research. Each of the procedures and analysis criteria are described in this section.

### Health needs

#### Classification of diseases

This study employs IPDs from the Global Health Estimates (GHE) cause categories established by WHO [46] according to the International Classification of Diseases, Tenth Revision (ICD-10). The GHE cause category has a hierarchical structure of four levels so that different levels of aggregation are included, as shown in Figure 11 in the Appendices. Three broad cause groups and 23 subgroups constitute level 1 and level 2, respectively: group I—communicable, maternal, perinatal and nutritional conditions (five subgroups); group II—noncommunicable diseases (16 subgroups); Group III—injuries (two subgroups). IPDs is a level-2 category under group I, which is the largest category of communicable diseases and contains 12 different conditions (level 3). Given the importance of accurate disease information for retrieving publication data, we excluded diseases with ambiguity (the 12th condition, “other infectious diseases” in IPDs) by referring to previous studies [30, 38]. Eleven different categories of IPDs are listed in Table 1 alongside a code we will use to refer to each condition in all subsequent tables and figures.

**Table 1:** Eleven specific conditions of IPDs

Code	IPDs
1	Tuberculosis
2	STDs excluding HIV
3	HIV/AIDS
4	Diarrhoeal diseases
5	Childhood-cluster diseases
6	Meningitis
7	Encephalitis
8	Hepatitis
9	Parasitic and vector diseases
10	Intestinal nematode infections
11	Leprosy

#### Burden of disease

Reliable and transparent health statistics are of great importance to anyone concerned with public health policy. To this end, a wide range of indicators have been developed to monitor and manage health initiatives. In line with similar prior studies [17, 21, 26], we take disability-adjusted life years (DALYs) to represent the burden of disease. DALYs are essentially a sum of the time lost through premature death plus time lived in a state of less than optimal health. In this sense, they quantify the difference between a person’s existing health status and ideal health conditions where one could expect to live to an advanced age, free of disease and disability [46].

To calculate the trends relevant to DALYs for different time stages, the DALYs estimates were collected from WHO for all categories of diseases for 183 countries in the years 2000, 2005, 2010 and 2015 (see Table 2). We then determined the burden of disease in terms of percentage of total DALYs for each country and each of the 11 conditions. To correct for differences in population size, we standardized the results as DALYs per 1000 population. In addition, we adopted the disease burden specialization index (SI) from Confraria and Wang [30] to reflect the “relative level of burden formulation in terms of the world-average level”, which is also free from the impact of population. This index can be expressed as:

1 $$\mathrm{S}{\mathrm{I}\_\mathrm{DALY}}_{rd}=\frac{{D}_{rd}/{\sum }_{d}{D}_{rd}}{{D}_{d}/{\sum }_{d}{D}_{d}}$$

where ${{\varvec{D}}}_{{\varvec{r}}{\varvec{d}}}$ is the number of DALYs in region ***r*** for disease ***d***, and ${{\varvec{D}}}_{{\varvec{d}}}$ is WHO’s estimated DALYs resulting from the disease ***d*** worldwide. $\mathrm{SI}\_\mathrm{DALY}>1$ indicates that region ***r*** carries a relatively higher burden from disease *d* than the world average, while $\mathrm{SI}\_\mathrm{DALY}<1$ indicates that this region carries a relatively lower burden from disease *d* than the world average. The value range of $\mathrm{SI}\_\mathrm{DALY}$ is null or positive infinity. Hence, this indicator is further standardized as the normalized SI (NSI), as follows:

**Table 2:** Data sources and periods

Period	I	II	III	IV	Source
DALYs	2000	2005	2010	2015	https://www.who.int/healthinfo/global_burden_disease/estimates_country_2000_2015/en/
Articles and reviews	2000–2004	2005–2009	2010–2014	2015–2019	Web of Science
HDI	2000	2005	2010	2015	http://hdr.undp.org/en/data

2 $$\mathrm{NSI}\_\mathrm{DALY}=\frac{(\mathrm{SI}\_\mathrm{DALY}-1)}{(\mathrm{SI}\_\mathrm{DALY}+1)}$$

Now, this measure has asymptotic limits of ±1, and a threshold value of zero.

### Research effort

Among the various types of academic research outputs, including academic publications, patents, and reports, peer-reviewed journals are the most common outlets for scholars to communicate about issues related to health [47]. Hence, academic articles have become the standard indicator by which research effort is measured when it comes to IPDs [38, 48].

We retrieved our corpus of articles from the Clarivate Analytics Web of Science (WoS) Core Collection using a search strategy developed by Confraria and Wang [30]. The strategy comprises a set of keywords strongly associated with different disease categories and 11 conditions of IPDs covered in this study. A more detailed explanation of the search strategy is provided in Appendix A1. We searched the title and keywords fields of WoS with a published date range of 2000 to 2019 and subsequently divided the results into four 5-year time periods (2000–2004, 2005–2009, 2010–2014 and 2015–2019). In total, we retrieved 393 716 articles and reviews. Note, however, that the sum of the 11 subcategories will be greater than this total since some articles belong to more than one disease category.

We further searched for all disease-related articles and reviews in the same period in WoS with a view to comparing the proportion of research effort devoted to IPDs versus the total burden of disease. It should be noted that the retrieval of all disease-related publication data also refers to the search strategy developed by Confraria and Wang [30]. Here, “all disease” refers to the sum of the GHE cause category’s five subgroups in group I and 13 subgroups in group II. As mentioned earlier, other subgroups in the GHE cause category are eliminated due to ambiguity.^2^ In total, we retrieved 2 387 505 articles from this search. To calculate the research effort, we again turned to Confraria and Wang [30], using their relative SI, expressed as follows:

3 $$\mathrm{S}{\mathrm{I}\_\mathrm{Pub}}_{rd}=\frac{{P}_{rd}/{\sum }_{d}{P}_{rd}}{{P}_{d}/{\sum }_{d}{P}_{d}}$$

where ${{\varvec{P}}}_{{\varvec{r}}{\varvec{d}}}$ is the number of articles produced in region ***r*** for disease ***d***, and ${{\varvec{P}}}_{{\varvec{d}}}$ is the number of articles related to disease ***d*** published worldwide. $\mathrm{SI}\_\mathrm{Pub}>1$ indicates that region ***r*** has exerted a higher research effort to address disease *d* than the world average, and $\mathrm{SI}\_\mathrm{Pub}>1$ indicates that this region has exerted a lower research effort to address disease *d* than the world average. A similar approach to the NSI of disease burden was also applied to normalize the $\mathrm{SI}\_\mathrm{Pub}$:

4 $$\mathrm{NSI}\_\mathrm{Pub}=\frac{(\mathrm{SI}\_\mathrm{Pub}-1)}{(\mathrm{SI}\_\mathrm{Pub}+1)}$$

For data visualization and analysis, we applied VOSviewer^3^ to generate a global collaboration network and visualizations on IPDs. VOSviewer is a software tool for constructing and visualizing bibliometric networks, developed by the Centre for Science and Technology Studies at the University of Leiden. VOSviewer works with bibliographic formats from WoS, Scopus, PubMed and RIS (Research Information Systems) files, among other. It can construct a visualization map through a co-occurrence matrix [49], which refers to collaborative relationships in this study.

The process of building the global collaboration network can be divided into three steps. In the first step, the country/region information is extracted from all publications and the number of collaborated publications for each country is calculated. Using the full counting approach, publications were assigned to a country according to the institutional address of each author [4]. In other words, an article was counted once for each country listed in the attributions. In the second step, the strength of collaboration is calculated by Salton’s measure [50], which is defined as the number of joint publications divided by the square root of the product of the total publications of the two countries (i.e. the geometric mean). And finally, in the third step, the co-occurrence matrix is constructed and inputted into VOSviewer to establish the global collaboration network. The final visualization is built by aligning the size of the nodes to the number of publications produced by the country, and the thickness of the link to the strength of the collaboration between the two countries.

It should be noted that in addition to the calculation of each country’s number of publications, the number of publications for different human development groups (countries/regions with various HDI levels) is counted directly on the aggregate level instead of summing the number of publications for each country/region within the specific HDI level, to avoid duplicate counting of publications with collaboration by countries/regions within the same HDI level.

### The HDI

A range of indicators or classifications have been developed to monitor and categorize a country’s development level, including the World Bank country classifications and the UNDP’s HDI. This study employs the HDI to classify countries into different development levels, since it serves as a more comprehensive measure of human development than purely economic measures [9]. The criteria used by UNDP and the World Bank to classify countries with varying levels of development are based on averages, with the key distinction being that the World Bank only considers the average per capita income, whilst UNDP considers overall development. Specifically, HDI is meant to be “a summary measure of average achievement in key dimensions of human development: a long and healthy life, being knowledgeable and having a decent standard of living”. The UNDP’s data centre describes the measure as “the geometric mean of normalized indices for each of the three dimensions”—health, education, and income [51]. The explanations are as follows:

*The health dimension is assessed by life expectancy at birth, the education dimension is measured by mean of years of schooling for adults aged 25 years and more and expected years of schooling for children of school entering age. The standard of living dimension is measured by gross national income per capita. The HDI uses the logarithm of income, to reflect the diminishing importance of income with increasing GNI [gross national income]. The scores for the three HDI dimension indices are then aggregated into a composite index using geometric mean.*

Based on the calculated HDI result, a country can fall into one of four categories—very high, high, medium and low. The HDI for each country is updated every year, with the exception of North Korea and Somalia, who do not have HDI indicators. Thus, we conducted our analysis on 181 countries, taking the HDI from the years 2000, 2005, 2010 and 2015 as our data. Table 2 summarizes the data sources and periods.

## Research effort and disease burden—results and analysis

The research framework in Fig. 1 shows that three different analyses are required to answer our research questions: an overall analysis, followed by analyses by country, and another tracing international collaborations. These are presented in the following three subsections, with the results both divided into periods and according to the four human development levels.

### Overall analysis

Note that all DALYs in this subsection are based on unstandardized total DALYs, because differences between population sizes are not relevant to these global results.

#### By IPDs' condition

**Fig. 2:**
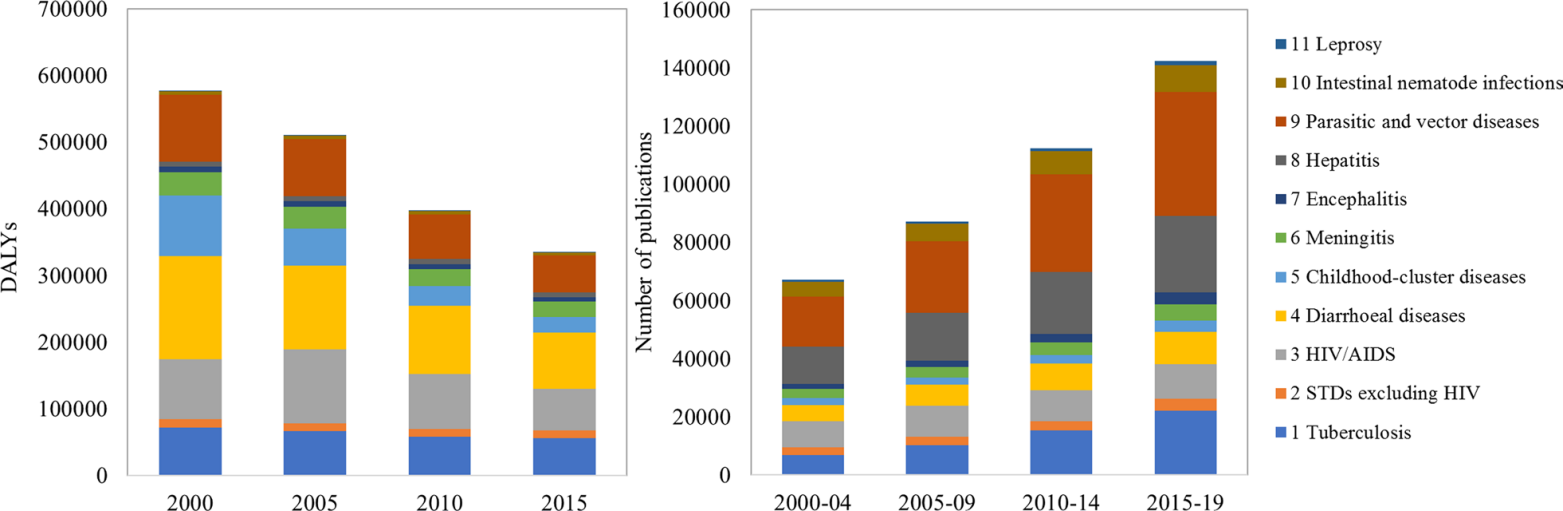
Disease burden and research effort by IPDs' condition in four periods

Figure 2 shows the disease burden and publication counts for the 11 subcategories of IPDs in four periods. With the steady improvement in health globally over the past 30 years [52], there has been a significant reduction in the global burden of IPDs in terms of DALYs. As for the effort side, the volume of IPDs articles shows a clear increasing trend over the past 20 years, as expected, with general growth in the number of publications and journal coverage in the WoS [53].

Overall, the publications and burden for 11 subcategories have both maintained relatively stable shares within the category of IPDs. Diarrhoeal diseases, parasitic and vector diseases, HIV/AIDS and childhood-cluster diseases are the four conditions with relatively high burden throughout all four periods. However, the distribution of publications for 11 subcategories is different from that of burden, in which parasitic and vector diseases, hepatitis, HIV/AIDS and tuberculosis represent a high number of articles.

Two diseases that are prominent on both the burden and effort sides are parasitic and vector diseases and HIV/AIDS. Parasitic and vector diseases accounted for the largest proportion of articles, at nearly 29% on average. A number of NTDs fall within this category, such as rabies, Chagas disease, leishmaniasis and schistosomiasis [54]. These are responsible for a significant disease burden in impoverished children and adults worldwide [55]. As for HIV/AIDS,^4^ it continues to be a major global public health issue, with an estimated 37.7 million people living with HIV at the end of 2020 [56]. Attention on HIV/AIDS was relatively high among the global scientific community, notably due to its incurability and the substantial and lasting negative social impact, especially in the hyperendemic countries of sub-Saharan Africa [57]. Moreover, as a condition strongly associated with HIV/AIDS, tuberculosis also accounts for a non-negligible share of publications on IPDs. Indeed, the weakened immune system caused by HIV greatly increases the risk of tuberculosis in people with HIV [58].

Hepatitis is the second most widely addressed condition in IPDs by academia. However, the burden caused by hepatitis seems to be inapparent globally. Hepatitis itself is an inflammation of the liver with five main strains, referred to as hepatitis A, B, C, D and E. However, it can progress to or be complicated by other diseases. In particular, types B and C are the most common causes of liver cirrhosis-, cancer- and viral hepatitis-related deaths in hundreds of millions of people [59], which has attracted extensive attention from academia. Further, China, as one of the high endemic geographical areas of hepatitis B virus (HBV) infection [60], had almost a third of the world’s HBV carriers in the early twenty-first century. With the high potential need regarding the prevention and treatment of this condition, relevant studies and trials were actively conducted by the Chinese scientific community, which might be one of the reasons for the high number of publications on hepatitis [61].

Diarrhoeal disease and childhood-cluster diseases are two subcategories with a remarkable burden but low level of research attention in IPDs. Diarrhoeal disease is the second leading cause of death in children under five years old and is mostly caused by contaminated food and water [62]. As a disease that is both preventable and treatable, it may be that alleviating the burden relies heavily on environmental improvement and access to treatment. Childhood-cluster diseases is a general term referring to several specific conditions, which are whooping cough, diphtheria, measles and tetanus. Among them, measles has the greatest burden, with more than 140 000 measles deaths in 2018 globally [63]. With the successful measles vaccine developed and improved in the 1960s, it has become one of the most cost-effective public health vehicles for preventing measles. Indeed, measles vaccination resulted in a 73% drop in measles deaths between 2000 and 2018 worldwide [64].

In addition, leprosy, an age-old disease with a treatment scheme comprising multidrug therapy (MDT) [65], constitutes the lowest proportion of publications on IPDs. In fact, the burden of leprosy is also the lowest in IPD. Encephalitis and sexually transmitted diseases (STDs) excluding HIV also account for a relatively low number of publications and burden.

In sum, this section examines the burden and publication volume in absolute terms for 11 IPDs. In the next part, a more extensive analysis will be provided in terms of the ratios of IPDs research/burden as a percentage of all disease-specific research/burden.

#### IPDs in comparison with all diseases

Here, the ratios of IPDs research/burden as a fraction of all disease-specific research/burden are firstly calculated and compared to observe the *relative* degree of scientific response on IPDs in comparison with all diseases globally. We also investigate the correlation between IPDs research efforts and disease burden.

**Fig. 3:**
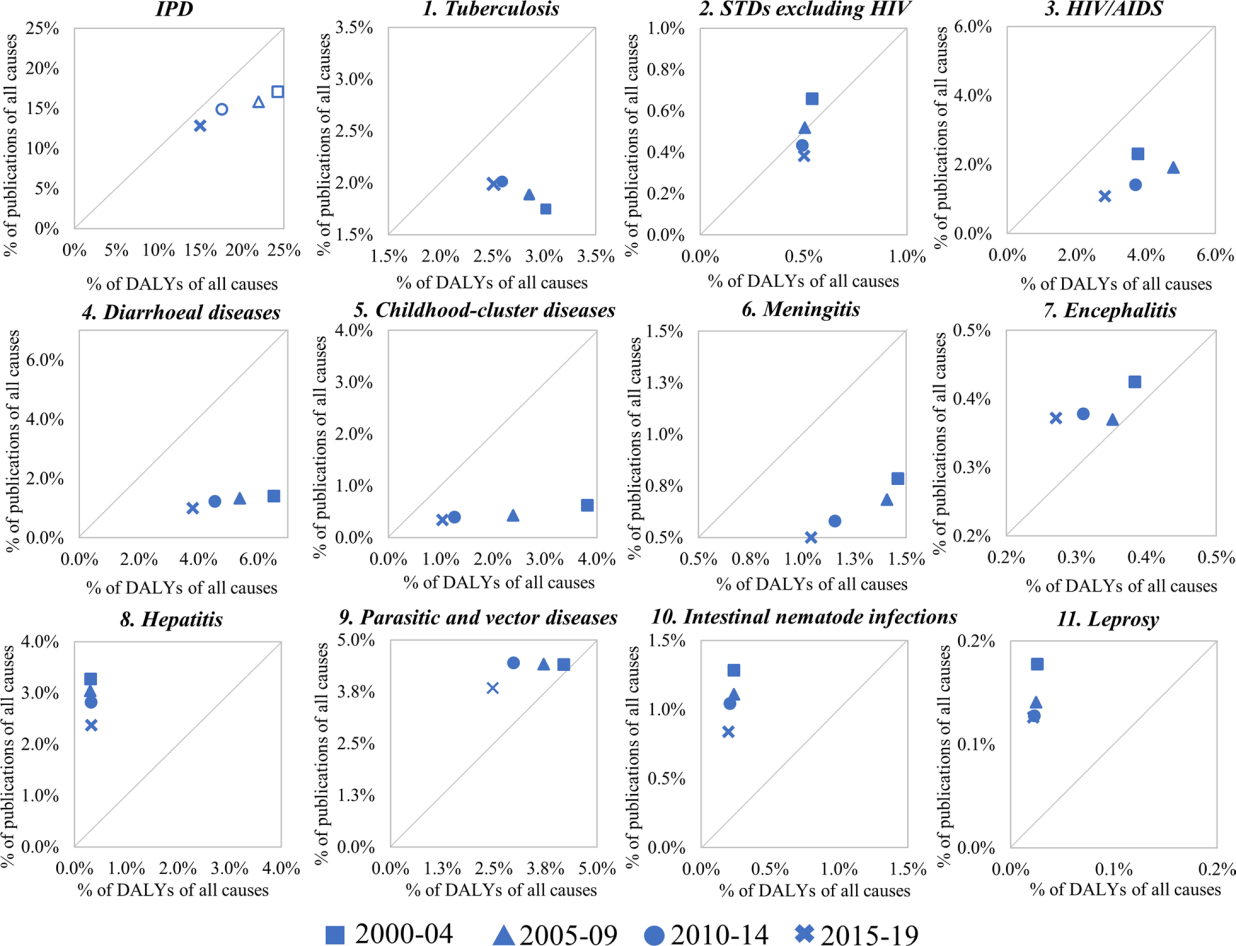
Research effort and burden of disease—IPDs versus all diseases

Figure 3 illustrates the distribution of publications and DALYs across the different time periods. Specifically, the share of disease burden (*x*-axis) versus the share of publications (*y*-axis) for the general IPD and its 11 subcategories is demonstrated by scatter plots. Here, the upper left corner of the diagonal line indicates where the research effort exceeds the disease burden and vice versa.

In the last two decades, the share of the general IPD's publication is lower than that of the burden they impose. Despite the steady increase in the number of publications on IPDs (Fig. 2), the share of its publications is decreasing over time (from 17.04 to 12.84%). On the burden side, a clear downward trend in the percentage of DALYs can also be observed (from 24.34 to 15.02%). The gap between research effort and disease burden, on the other hand, does seem to be narrowing, as also revealed by the continuously approaching positions with respect to the diagonal line of the general IPD in Fig. 3.

In terms of the 11 subcategories, the percentage of DALYs for most conditions is decreasing, with the exception of hepatitis. The percentage of DALYs of hepatitis remained consistent during 2000–2019, and even climbed marginally. Interestingly, despite the fact that its global burden appears to be insignificant, hepatitis is the disease with the second highest number of publications, trailing only parasitic and vector diseases, implying that diseases with stable burden have received greater attention as the overall burden of IPDs has decreased. Combined with the above discussion on hepatitis, the potential for complication by other diseases, the active involvement of Chinese academia in relevant research and its stable burden might be the reason for the significant scientific attention on hepatitis.

On the research effort side, corresponding to the general decreasing trend in the share of IPDs publications, the proportion of publications declined throughout the 11 subcategories, except for tuberculosis. Among the 11 subcategories, tuberculosis represents the fourth most severe burden of infectious disease worldwide. Furthermore, among the top five high-burden infectious illnesses, the burden of tuberculosis is dropping at the slowest rate. At the national level, tuberculosis remains a serious health concern in certain populous nation, such as India and China, which have also made significant contributions to related research. In addition, the previously indicated link between tuberculosis and HIV/AIDS is another possible explanation for the growing volume of literature on the disease.

Among the 11 subcategories, diarrhoeal diseases, childhood-cluster diseases and meningitis are diseases with a steady decline in both burden and share of publications. In particular, the burden of diarrhoeal diseases and childhood-cluster diseases was substantial in 2000, but has declined dramatically in subsequent years. While the proportion of publications remains lower than the percentage of DALYs for both diseases, the gap between the share of publications and the burden is closing. Encephalitis, hepatitis, parasitic and vector diseases, intestinal nematode infections and leprosy were the five diseases with greater publication share than burden. Except for parasitic and vector diseases, the burden of these diseases is fairly low. The various reasons for the large number of publications on hepatitis have already been discussed. The absolute number of publications for the remaining three diseases is equally low when compared to infections with a high burden. Parasitic and vector diseases is prominent on both the burden and effort sides. This category contains a variety of NTDs, which are not only responsible for considerable burden, but also have garnered increased attention from the global scientific community [66]. Furthermore, previous study has verified that the United Kingdom, a highly developed country, has sponsored a large number of studies on high-burden diseases in less developed areas, especially on parasitic and vector diseases [17].

**Fig. 4:**
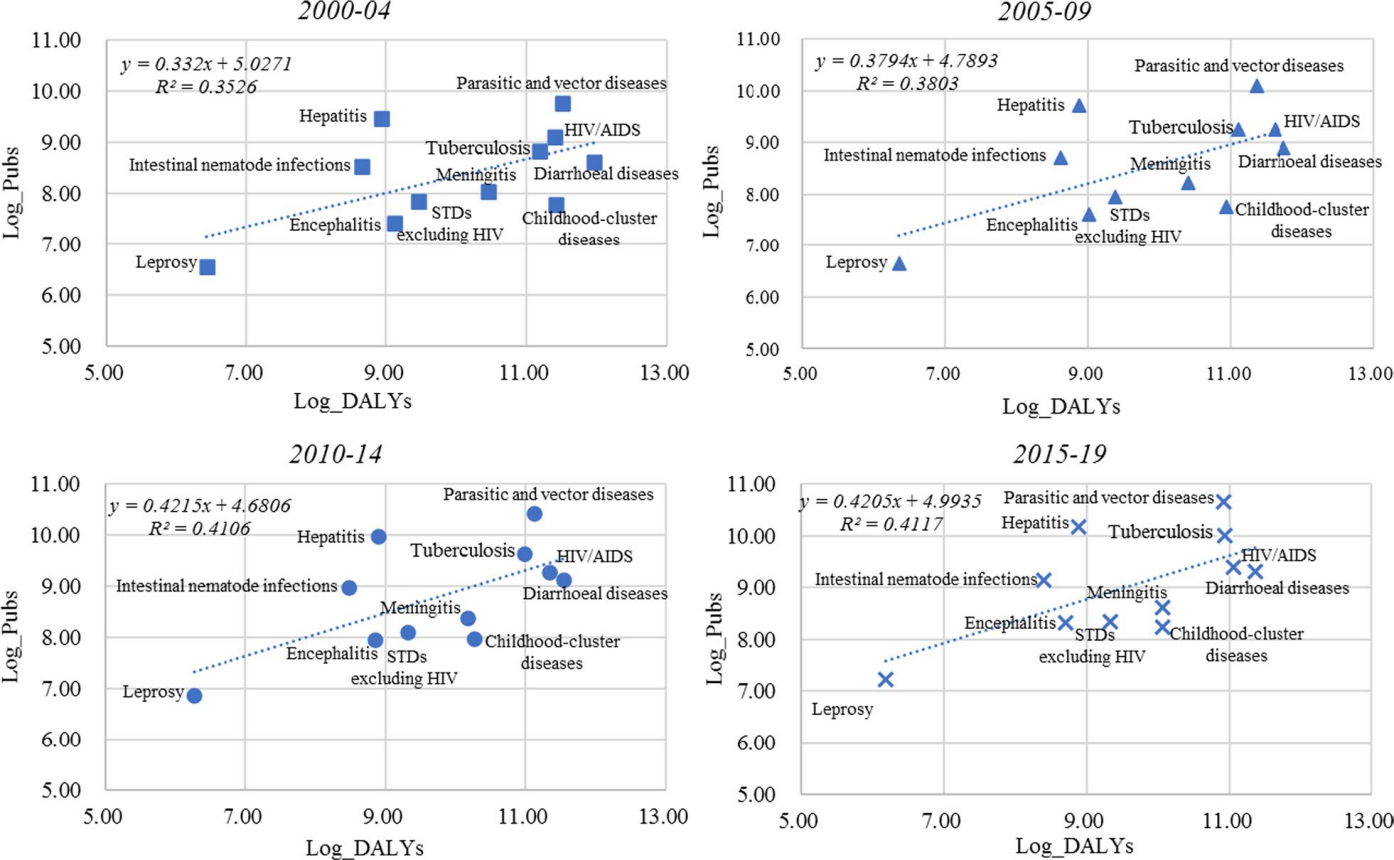
Disease burden (log) versus research effort (log) by IPDs' condition

Figure 4 displays the logarithms of research effort versus the burden of disease for each of the 11 IPD subcategories in four periods. All graphs indicate a positive correlation, which means that, on average, the higher the disease burden, the greater the number of articles published. As revealed by the increasing correlation coefficient for the four periods in general, research efforts on IPDs tend to increase as time passes. Overall, although the research efforts on IPDs tend to decline as a share of all causes (Fig. 3a), academia has generally responded positively to health needs caused by IPDs.

### By country

#### Overall

As expected, the countries with the highest disease burden are mainly concentrated in the less developed regions of Africa, with all top 40 countries with the greatest disease burden (DALYs standardized per 1000 population) situated on this continent. However, within these 40 countries, the particular conditions imposing the highest burden vary from country to country. Taking the top five as an example (Sierra Leone, Central African Republic, Malawi, Zimbabwe, Lesotho), HIV/AIDS tops the list in Malawi, Zimbabwe and Lesotho, while in Sierra Leone and the Central African Republic, HIV/AIDS is nudged into lower place by parasitic and vector diseases and also diarrhoeal diseases.

**Fig. 5:**
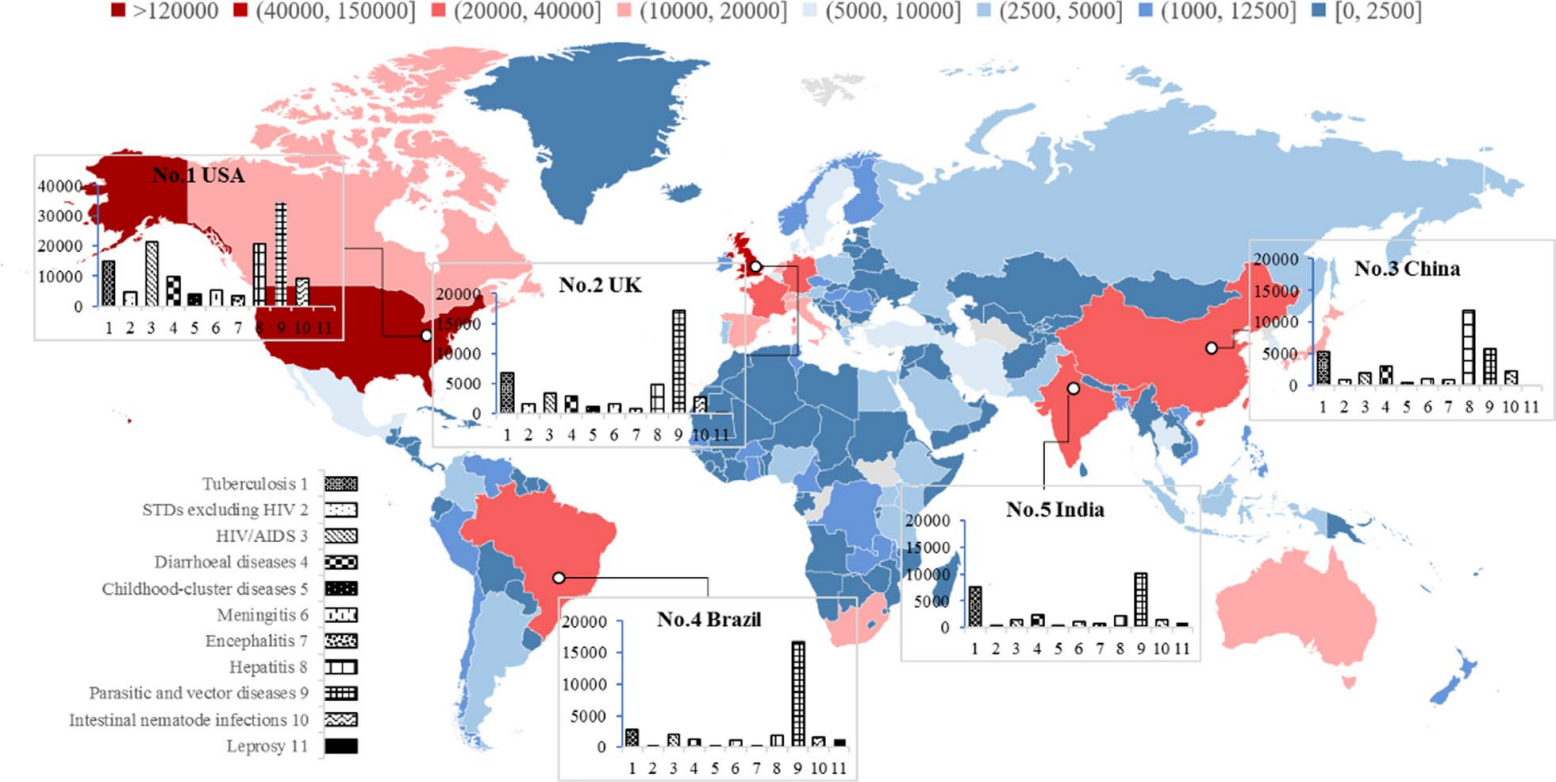
The geographical distribution of research effort—top five countries

The countries with the highest academic contributions are dispersed throughout the developed continents of the Americas, Europe, Asia and Oceania. Moreover, the top three countries with significant contributions are the traditional forces on academic publishing worldwide. Figure 5 shows the geographical distribution of research effort in the 11 different conditions. Obviously, the United States leads the world, with an absolute advantage of almost 130 000 publications, followed by the United Kingdom, China, Brazil and India. All these countries have relatively low burden of IPDs, ranking outside the top 100 list on DALYs per 1000 population globally, except for India (ranked 55th).

What is noteworthy is that parasitic and vector diseases is the most attended to category in the countries with high publication volumes, except for China. This type of IPDs carries one of the highest burdens for Sierra Leone and the Central African Republic, while research effort from China emphasizes diseases with a high potential local burden, as mentioned above, such as hepatitis. HIV/AIDS has received substantial attention from scholars in the United States and United Kingdom. Tuberculosis has also been a subject of interest for the top five publishing countries.

The tendency of developed countries to give attention to global health needs was verified in a prior study on the funding priorities of public funding agencies in the United Kingdom and China [17]. Their results indicated that the United Kingdom funds a wider variety of research, extending to projects with impacts outside its borders and, among these, to some developing countries. Additionally, both public and nonpublic funding institutions in developed countries, such as the National Institutes of Health and the Bill & Melinda Gates Foundation in the United States, have established priority funds for research into the infectious diseases affecting the poorest regions and populations [67, 68].

#### By human development level (absolute global share)

Note that the following is a dynamic analysis over 20 years, during which the development level of many countries is constantly changing. Hence, we chose to divide countries according to their dynamic HDI levels to reflect the research response pattern of regions with four HDI levels globally against IPDs. Further observation and interpretation of “fixed-level” countries and others with a high impact on the results of global shares will also be provided in this section. Table 3 shows that the number of countries falling into the low and medium development groups is decreasing over time. Accordingly, countries are increasingly meeting the threshold for classification as high and very high HDI.

**Table 3:** Number of countries at different HDI levels in each of the four periods

HDI	2000–2004	2005–2009	2010–2014	2015–2019
Low	58	55	46	39
Medium	58	44	41	38
High	34	42	47	48
Very high	31	40	47	56

Table 4 lists the disease burden and research effort for each of the four HDI groups in each period, in which the proportion of the burden and publications of countries/regions in a given HDI level is calculated relative to the global burden and total number of publications, respectively. Disease burden is shaded in green, with darker shades representing a greater burden of disease. Research effort is shaded in yellow, where, again, the darker the shade, the greater the research contribution.

**Table 4:**
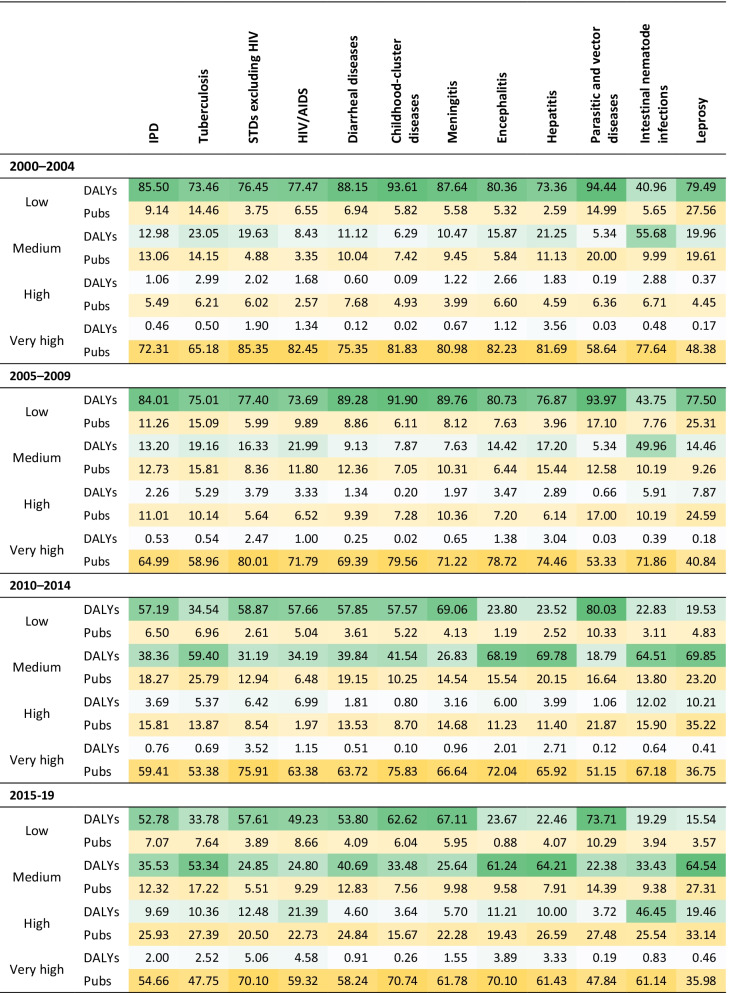
Global share of research effort and disease burden by human development level

DALYs are unstandardized. IPD is the aggregate total of the 11 subcategories

Consistent with the design of the HDI, countries in the lowest human development group carry the greatest disease burden. Likewise, the well-resourced infrastructure of countries in the very-high development group is responsible for contributing the most research to the literature. However, despite a steady increase in the number of publications produced by the very-high group across the four periods, the proportion of research effort they contribute is declining. In other words, the number of articles on IPDs produced by the other groups is also increasing and at a greater rate. In fact, the proportion of publications generated by those traditional highly developed countries (i.e. 31 countries in the very-high group in all four time periods) declined in a more significant manner from 72.31 to 48.29% (see Table 5). In contrast, the contribution of countries *always* in the low HDI group (i.e. 39 countries in the low group in all four time periods) showed a clear and substantial increase (from 3.63 to 7.07%), which speaks to a persistent effort by scholars in the areas affected to do something to ease their high local burden.

**Table 5:** Global share of research effort and disease burden for *always* low and very-high group

		IPD	Tuberculosis	STDs excluding HIV	HIV/AIDS	Diarrheal diseases	Childhood-cluster diseases	Meningitis	Encephalitis	Hepatitis	Parasitic and vector diseases	Intestinal nematode infections	Leprosy
2000–2004													
Low	DALYs	51.95	26.48	41.17	48.25	46.33	64.72	58.14	19.09	19.27	78.04	13.36	12.76
	Pubs	3.63	4.92	1.76	0.90	1.37	2.82	2.15	0.29	0.57	7.41	1.44	6.62
Very high	DALYs	0.46	0.50	1.90	1.34	0.12	0.02	0.67	1.12	3.56	0.03	0.48	0.17
	Pubs	72.31	65.18	85.35	82.45	75.35	81.83	80.98	82.23	81.69	58.64	77.64	48.38
2005–2009													
Low	DALYs	51.13	28.44	46.40	45.83	47.97	61.26	63.71	21.12	20.87	77.34	15.55	13.60
	Pubs	4.35	4.29	2.85	5.39	1.82	3.52	2.71	0.48	1.36	8.24	2.03	4.12
Very high	DALYs	0.47	0.43	2.19	0.89	0.24	0.02	0.57	1.22	2.92	0.03	0.38	0.18
	Pubs	61.74	58.07	78.45	71.38	67.87	78.66	70.15	78.20	73.52	52.81	70.30	40.53
2010–2014													
Low	DALYs	51.01	30.17	52.63	47.79	51.56	52.86	63.39	20.29	21.23	75.43	17.70	14.94
	Pubs	5.64	6.16	2.45	2.66	3.36	5.09	3.83	0.46	2.42	9.35	2.62	3.39
Very high	DALYs	0.53	0.38	2.50	0.83	0.42	0.03	0.61	1.42	2.34	0.03	0.38	0.19
	Pubs	55.02	51.22	73.10	63.68	60.76	73.46	64.24	68.55	63.61	49.00	63.48	35.99
2015–2019													
Low	DALYs	52.78	33.78	57.61	49.23	53.80	62.62	67.11	23.67	22.46	73.71	19.29	15.54
	Pubs	7.07	7.64	3.89	8.66	4.09	6.04	5.95	0.88	4.07	10.29	3.94	3.57
Very high	DALYs	0.59	0.36	2.48	0.89	0.60	0.03	0.59	1.55	2.23	0.04	0.36	0.21
	Pubs	48.29	43.55	65.21	56.55	51.80	65.69	57.56	63.15	55.48	44.32	55.76	30.48

DALYs are unstandardized. IPD is the aggregate total of the 11 subcategories

Special attention is given to countries in the always-low HDI level, who are also the ones with high burden of IPDs, to further observe their scientific response pattern to local burden. In addition to the constantly increasing number of publications mentioned above, the distribution of research efforts from the always-low group on the 11 specific conditions also corresponds to the distribution of burden in those regions to some extent, as shown in Table 5. In particular, parasitic and vector diseases caused the highest burden among all 11 conditions in terms of both original values and global shares for the always-low group. Correspondingly, publications on this disease accounted for the highest proportion for those undeveloped regions. Moreover, although the parasitic and vector disease category is already the category of highest concern among the 11 diseases by scholars around the world (Fig. 2), the proportion contributed by the always-low group with respect to the whole world still increases from 7.41 to 10.29%, as Table 5 shows. In spite of the general growing share of publications on IPDs, leprosy is the only disease with a sustained decline in the proportion of publications in the last two decades for the always-low group, which is also the one causing the least burden in those regions. In sum, those undeveloped areas have contributed concentrated and specialized efforts to ease their local burden. In the context of the growing literature on IPDs globally, the proportion of publications contributed by those underdeveloped regions is increasing remarkably. As for the burden side, although the global share of DALYs for the always-low group fluctuates slightly around 52% (Table 5), an obvious downward trend in DALYs original values for those regions can be observed over the last two decades.

A closer look at specific countries behind the HDI groups might provide deeper insight into how the least developed countries defend themselves against the threats of infectious diseases. Indeed, 6 of the 10 African countries with the highest number of publications are consistently classified in the low HDI group. Specifically, four of the six countries are still on the latest United Nations list of least developed countries [69]. Among them, Uganda has the largest publication output, and also bears a high burden of IPDs (ranked eighth and ninth of DALYs in original value and per 1000 population, respectively). Hence, Uganda is chosen as a typical case in this section. With the continuously growing proportion of publications on IPDs, Uganda’s global rank for DALYs in both original value and per 1000 population declined significantly from 2000 to 2019, illustrating a greater reduction in IPDs burden compared with the overall global decline. In terms of the specific diseases, parasitic and vector diseases and HIV/AIDS are the two major causes of such high burden in Uganda. Accordingly, among IPDs publications produced by Uganda, almost 70% targeted these two diseases. Indeed, Uganda has been widely regarded as one of the world’s earliest and most compelling national success stories in combatting the spread of HIV [70, 71], experiencing the sharpest decrease in HIV/AIDS-related death globally between 1990 and 2017 [72]. In addition to the massive efforts on scientific research, Uganda has established a comprehensive HIV/AIDS programme with high-level political support and multisectoral response [73]. Indeed, both the scientific research and policy implementation is inseparable from international assistance. Early in 1986, the representative from Uganda at the World Health Assembly (WHA) informed the assembly of the domestic HIV/AIDS endemic and called for the support and help of the international community [74]. Its high dependency on international collaboration can be seen in the high share of international collaborative IPDs publications (90.26%), especially with highly developed countries (i.e. 50.41% of publications are collaborations with the United States). As for the parasitic and vector diseases, corresponding to the steady rise in the number and global share of publications targeting this disease, Uganda’s burden decreased markedly in terms of both global share (from 5.18 to 2.48%) and ranking (from 4th to 11th).

In addition to the conspicuous patterns of the very-high and low groups, the shares of publications and burden for high and medium group seem like to fluctuate more erratically. Generally speaking, in all periods except the most recent, the proportion of publications produced by countries in the high group was even lower than that in the medium group. Obviously, medium-group countries carry a greater burden of IPDs, and so have a stronger incentive to address the subject. Another possible explanation can be found by further digging into the data—China moved from the medium group into the high group in the last period. With the full measure of China’s output counted in the medium bracket up until then, it is no wonder that high-group countries could not compete. Indeed, the medium and high levels are the two with the most frequent mobility in countries. The change in HDI levels of countries with high absolute value of disease burden (and large populations) and publication volume will greatly affect the results of these two levels.

In particular, India, a country with a large population, has the highest original DALYs across the whole study period on the aggregate IPDs level. Hence, the upgrade of India from the low to medium level in the third period inevitably leads to the increasing share of burden for medium groups, especially for several diseases with the highest burden in India (tuberculosis, encephalitis, hepatitis and leprosy). Moreover, China also has a high relative value of original DALYs in encephalitis and hepatitis (both ranked among the top five globally). Indeed, although China’s burden of those two diseases is lower than India’s, the large volume of publications generated by Chinese scholars, together with China’s movement from the medium to high group in the last period, resulted in the growth in the share of publications of the high group. As for intestinal nematode infections, China ranked first in original DALYs from 2000 to 2019, which explains the high global share of the burden in the medium group during 2000–2004 and its substantial increase in the high group in 2015–2019. Correspondingly, the intensive effort from China on intestinal nematode infections, especially in the latest period, contributes to a marked rise in share of publications for the high group in 2015–2019. Unlike the above-mentioned diseases, the increased share of both burden and publications for HIV/AIDS in the high group in 2015–2019 is mainly due to the upgrade of South Africa from medium to high group in the same period. On average, South Africa ranked first and third in burden and publications, respectively, which also partly reflects its high research attention on local health needs.

In spite of the highest share of burden and publications in the low and very-high groups, respectively, the tendency to give priority to local health needs can be observed not only from the steady increase in the always-low group’s share of publications, but also from the intensive research efforts on disease with high local burden from several major countries with mobility in HDI levels. This conclusion, however, does not consider the effort a country extends towards IPDs as a proportion of its entire disease-related research. Thus, one may reach a different conclusion when examining how a nation balances its research efforts between local and global needs. Such analysis needs to be based on a country-specific perspective, combined with the information of burden and research effort for all categories of diseases instead of only IPDs. This is somewhat beyond the scope of this paper, as we are focused on countries’ research efforts on IPDs, not the specific research priorities of each country. That said, this reasoning raises the point that our results cannot be taken at face value. Rather, they must be interpreted more carefully from a multidimensional and dialectical perspective. Indicators such as the percentage of a country’s publications devoted to IPDs relative of that of the world cannot accurately reflect the true dimensions of specific countries’ research concerns. A more fine-grained analysis is needed to address this problem.

#### By human development level (relative SI)

**Fig. 6:**
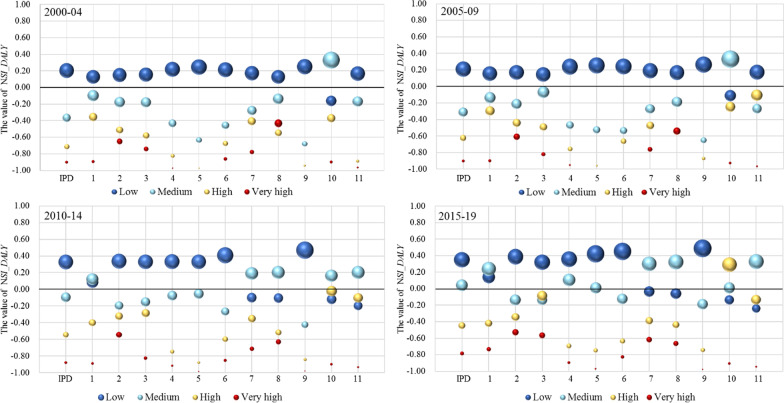
$\mathrm{NSI}\_\mathrm{DALY}$ by human development group. IPD is the aggregate total of the 11 subcategories. The category names for codes 1 to 11 are given in Table 1. The size of the bubbles represents the disease burden

**Fig. 7:**
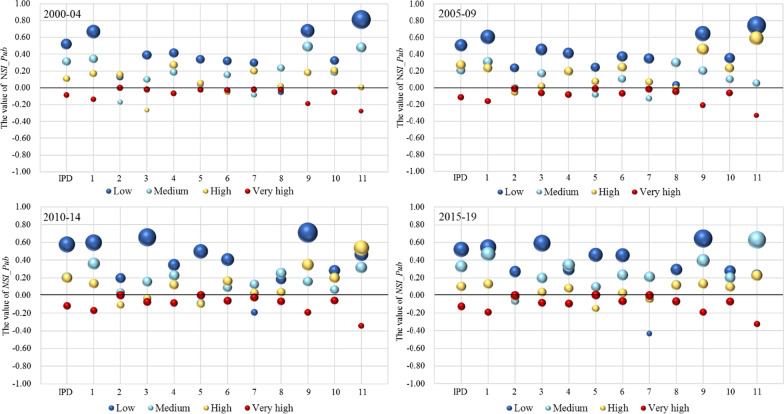
$\mathrm{NSI}\_\mathrm{Pub}$ by human development group. IPD is the aggregate total of the 11 subcategories. The category names for codes 1 to 11 are given in Table 1. The size of the bubbles represents the research effort

Following the global share of disease burden and research effort calculated based on original absolute numbers, this section adjusts the two factors to reflect *relative* level of burden and efforts with respect to the world average without the impact of population size. Figures 6 and 7 show the disease burden and research effort, as calculated by the normalized SI (NSI) for the different human development groups.

The results for disease burden (Fig. 6) are similar to the previous analysis, with countries in the low group carrying a greater burden than the world average. Moreover, over the full period, this tendency has only strengthened, with the value of $\mathrm{NSI}\_\mathrm{DALY}$ increasing for both low and medium groups. So, although the burden of IPDs has decreased overall, the problem for many low- to medium-HDI-level countries is still serious. One possible explanation for an increased burden of disease in the medium group is that more than 10 countries that were previously in the low group had their status upgraded in the later periods. It would not be surprising to find that IPDs have not been alleviated even if other indicators of human development have risen. Several diseases in Fig. 6 show a particular tendency to shift from low to medium and even to the high groups, which are basically the same as those discussed above—encephalitis (7),^5^ hepatitis (8), intestinal nematode infections (10) and leprosy (11).

Figure 7 shows the research effort for different groups, where we find the patterns of the previous analysis amplified—low-level countries are clearly exerting the greatest effort in research on IPDs, with the only exception of encephalitis (7). As mentioned, the overall number of publications on encephalitis (7) is the second lowest among all 11 conditions, in which India contributed the majority for the low group in the first two periods. Nevertheless, a strong conclusion drawn from this result is that low-level countries are consistently striving to ease their own burden. Another notable point is that the value of $\mathrm{NSI}\_\mathrm{Pub}$ for the high group was higher than zero in most cases. This means that those countries appear to be expending more effort to explore IPDs than the world average.

What seems contrary to the above analysis is that the very-high group is making the least research contribution (see red bubbles in Fig. 7). In examining the data behind these averages, almost every country made less effort than the global average. However, even at the least effort, the number of articles published absolutely eclipses any other group. For all diseases, very-high-level countries generate 75.7% of the total articles. For IPD-related articles, that number is 60.6%. In other words, in absolute terms, the world average is highly influenced by the very-high group. Hence, the $\mathrm{NSI}\_\mathrm{Pub}$ of the very-high group will naturally fall below zero when low-group countries concentrated more on IPDs than on other types of disease within their research capability.

We now turn to the balance of research effort between global and local needs. Since low-level countries are exerting such a strong effort to research IPDs, we can also surmise that they are prioritizing local issues. In terms of the other three groups, it is difficult to argue any firm conclusions without a thorough and detailed exploration of the other disease burden they may be facing. However, as the figures for research effort across all diseases show (see Appendix A2), the very-high group tends to focus more on noncommunicable diseases, such as neurological conditions and cardiovascular disease. These are diseases of high burden in developed countries [52].

The tendency to focus more on local health needs was verified for both developed and undeveloped regions in the above analysis. Again, however, we must highlight that an integrated exploration of burden and research efforts on all types of disease for specific countries is needed to reflect detailed information on the balance between global and local needs. Nevertheless, what clearly emerges from this analysis is the complexity of setting research priorities and establishing a research agenda by referring to quantitative indices. Taking our results as an example, if one relies only on the results produced by the absolute calculations, then practical issues such as the actual research capacity of each country might be overlooked. Under such circumstances, analysing the quantitative outcomes from multidimensional perspectives and integrating them with qualitative analysis may help in developing more rational and effective policies.

### International collaborations

This section provides an analysis of the collaboration patterns in two respects: overall and with respect to the countries with a high disease burden.

#### Overall

**Fig. 8:**
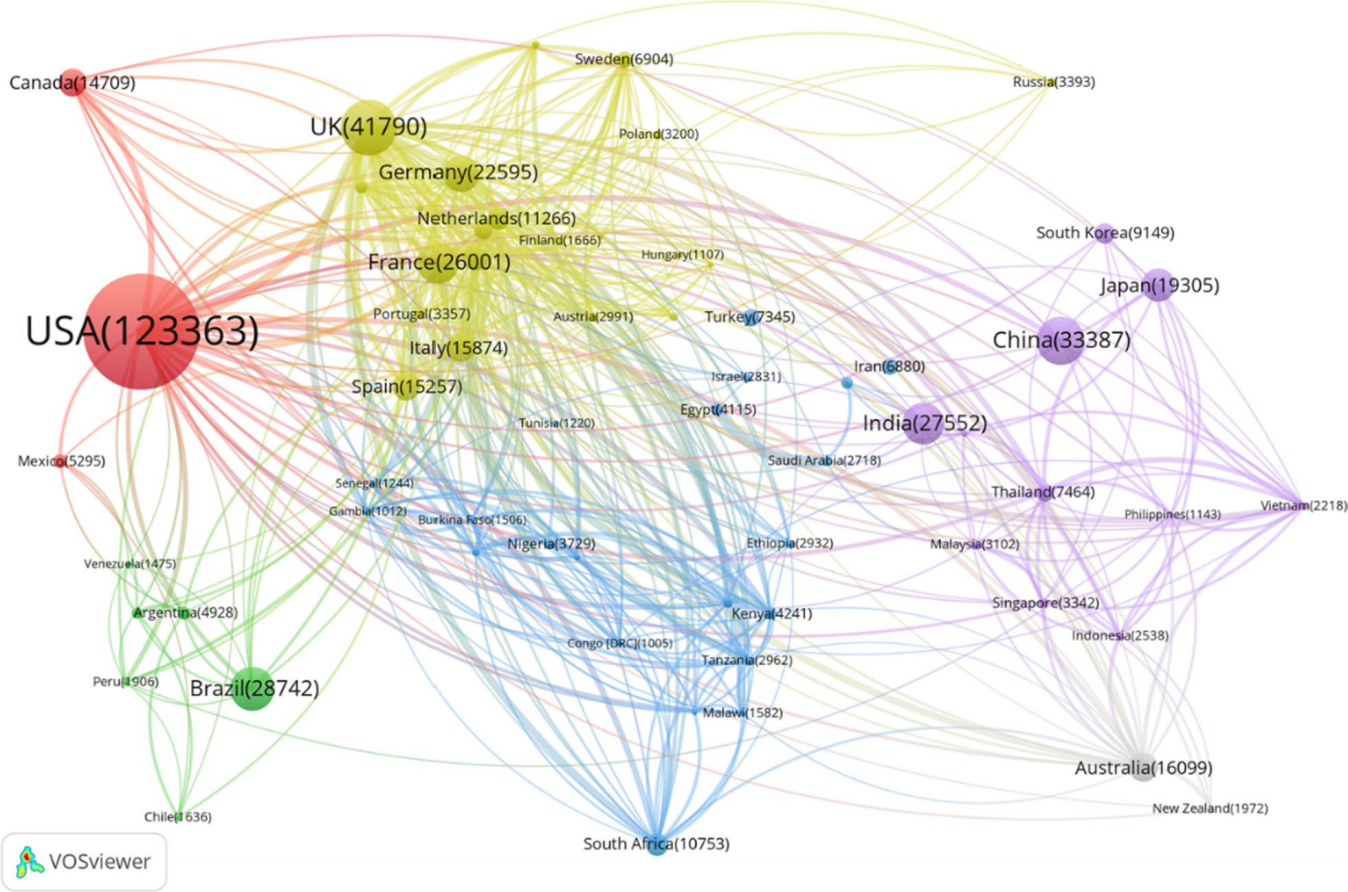
International collaboration patterns. The size of the node indicates the number of publications produced by the country, which corresponds to the number presented on the node. The thickness of the link reflects the strength of the collaboration between the two countries. The different colours relate to the continent a country is situated on

Among all the countries that have been involved in scientific publishing on IPDs, 65 have generated more than 1000 publications, and these we selected as the major subjects of study in this section. Figure 8 shows a collaboration network of these 65 countries, visualized through VOSViewer [49].

Collaborations occur on all continents,^6^ especially in Europe. With the largest publication total, the United States has a significant number of collaborations with countries from all the other continents. Scholars in the United Kingdom work closely with researchers both in neighbouring countries and in Africa to tackle the public health needs raised by IPDs. Although ranked third for research effort, China generated many fewer links than the United States and United Kingdom, and Chinese scholars seem more inclined to collaborate with scholars from other Asian countries. What is promising is that in Africa, 21 countries have published more than 1000 papers, of which South Africa leads the list with more than 10 000 papers. Further, intensive collaborative studies have also been conducted within the African continent and with the West.

**Fig. 9:**
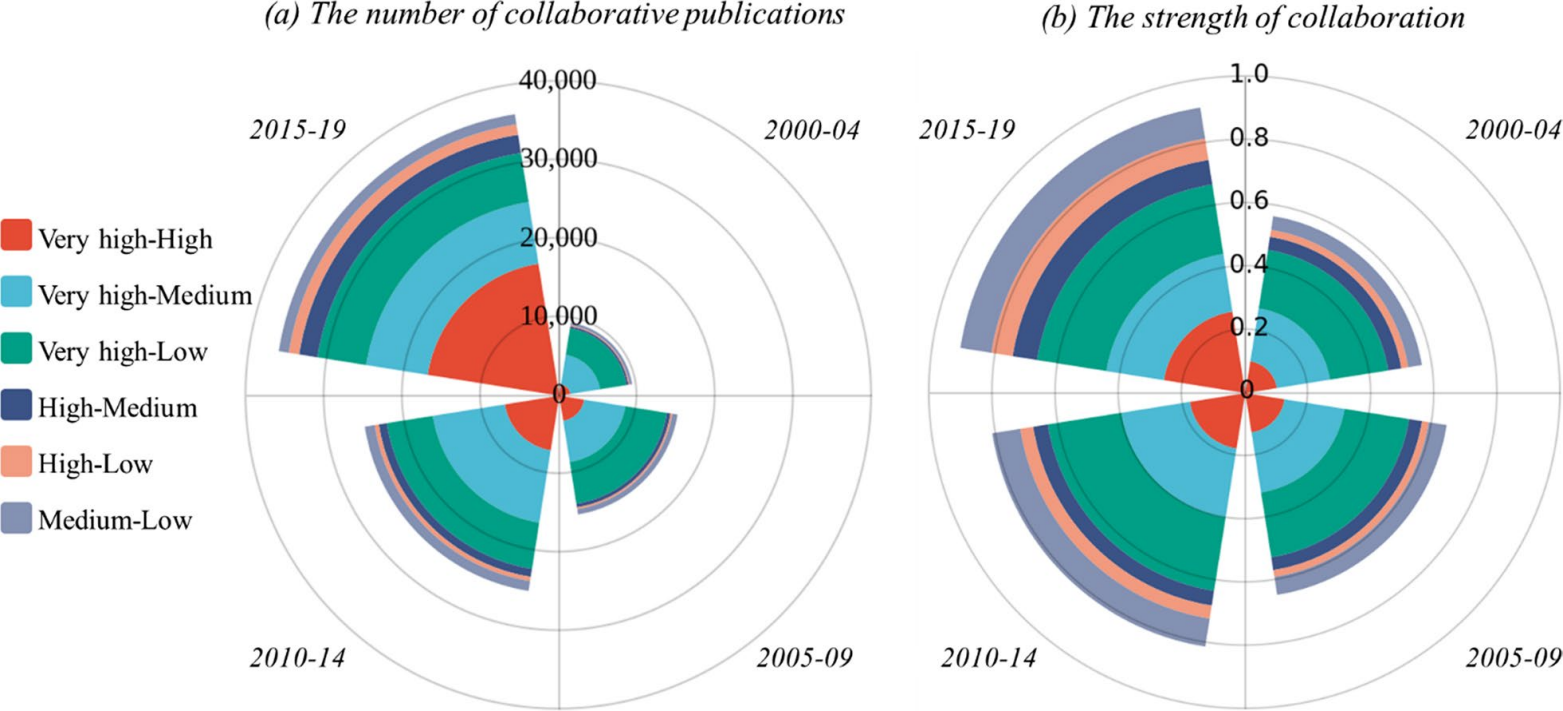
Collaboration patterns between HDI groups by time period

In order to further explore the variation trends in collaboration patterns worldwide, the number of collaborative publications and collaboration strength are calculated for six collaboration pairs generated by four HDI groups in different time periods, as shown in Fig. 9. Here, each coloured annular sector represents a collaboration pair, and the distance between the outer arc length and inner arc length of each sector depicts the number of collaborative publications or collaboration strength for that pair. The colours and corresponding pairs are given on the left side of Fig. 9. From a comparative perspective, as expected, the most collaborative countries are those in the group with very high human development. This group has three country pairs. However, collaboration strength is highest between the very-high and the low group, although the other two pairs have a larger number of collaborative publications. It is worth noting that the collaboration between very high and high countries intensified significantly post-2015 when China shifted groups. High–low pairs were the least cooperative.

From the perspective of time evolution, both the number of collaborative publications and the strength of the collaborations increased over the period, especially for the very-high and high group collaboration pair (the red sector). With the general increase in the number of IPDs publications, it is quite natural to observe the growing volume of collaborative publications. Furthermore, one possible explanation for such an increasing trend in the very-high and high group pair is the advancement of the countries’ development level. For instance, three productive countries (Brazil, China and South Africa) moved into the high group in various periods. Their primary collaborators are all from the very-high group, which increases the number of joint publications and the strength of collaboration between very-high and high groups. At the same time, the strength of collaboration between the low and medium groups has grown as a consequence of an increase in the number of joint publications by the two groups but a decline in the number of their individual publications. Specifically, China’s transition from medium to high group and India’s shift from low to medium group resulted in a decrease in publication volume in medium and low groups. However, the number of collaborative publications between the two groups continues to increase, reflecting the less developed regions’ intensive collaboration in responding to their local needs.

As for the closer collaborative relations observed among different collaboration pairs, further observations are made from the perspective of number of HDI groups involved in one publication. The ratio of publications generated within the same HDI group^7^ experienced a steady decline from 86.46 to 78.27% over the four time periods, indicating the increasing proportion of cross-HDI-level collaborative publications. Specifically, the ratios of bilateral collaborative publications generated between two HDI groups for the six pairs also show general downward trends from 2000–2004 to 2015–2019. Obviously, the more frequent multilateral collaboration among three or four HDI groups also contributes to a higher value of collaboration strength in the later period.

#### With respect to disease burden

**Fig. 10:**
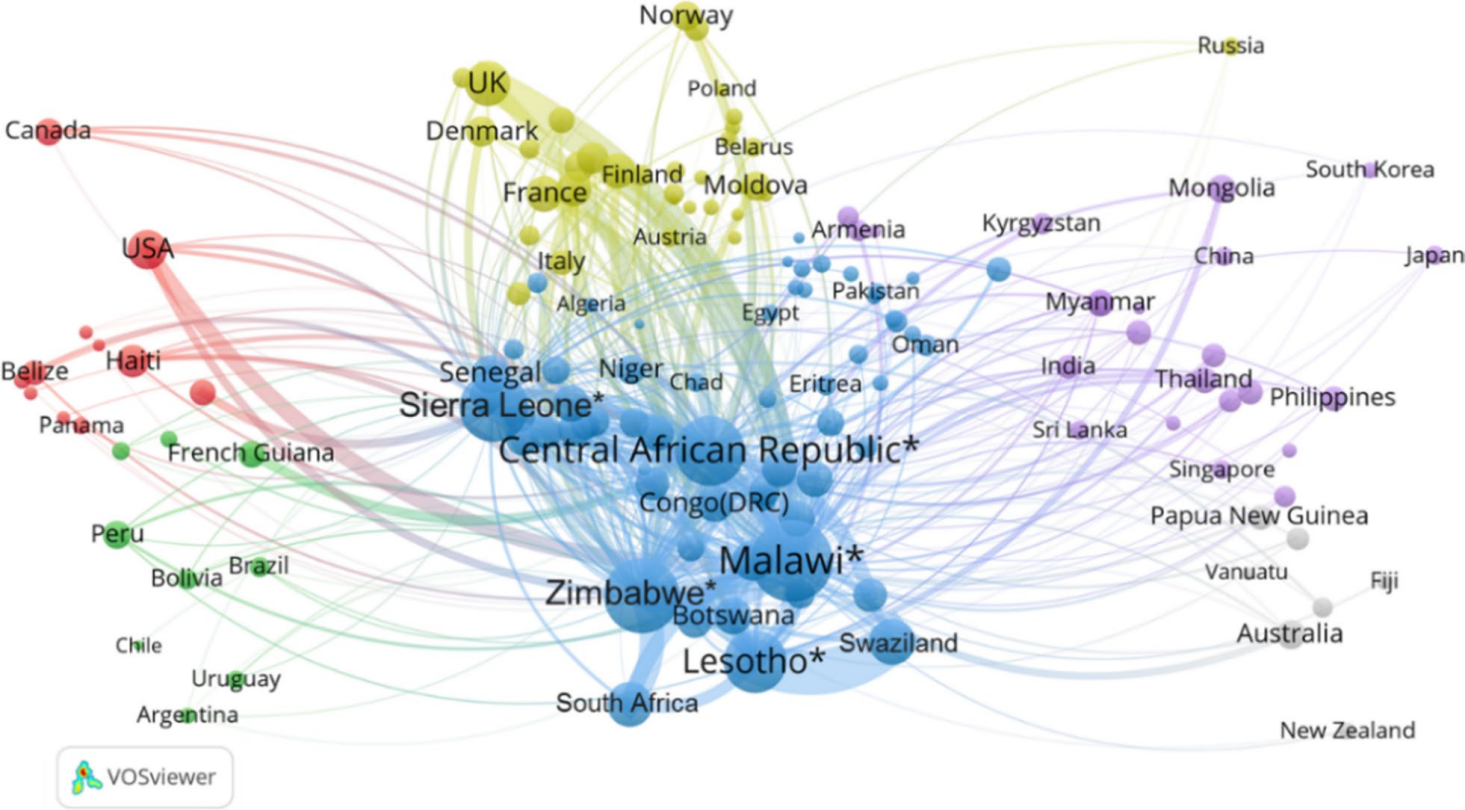
Collaboration network of the five countries with the greatest disease burden. The size of the node indicates the number of other countries a nation collaborates with. The thickness of the link reflects the strength of the collaboration between the two countries. The different colours relate to the continent a country is situated on. *Indicates countries with the highest burden

**Fig. 11:**
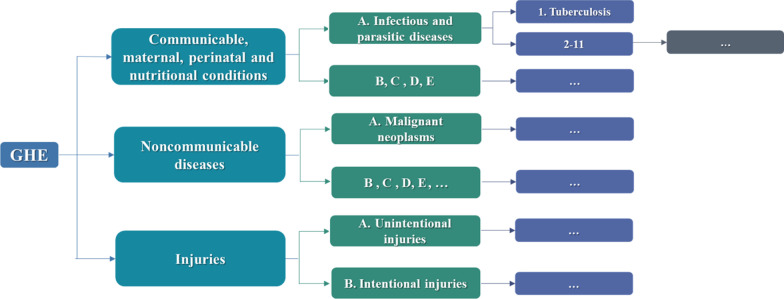
The hierarchical Global Health Estimates (GHE) cause category

As mentioned, high-burden countries are concentrated in Africa. However, among the 21 countries on the African continent with more than 1000 publications, only Malawi and Nigeria are in the top 10 list of countries with the greatest burden of IPDs, ranked third and sixth, respectively. To gain further insight into how countries with the highest IPDs burden conduct relevant research, the five countries with the highest burden (countries with * in Fig. 10) are regarded as targets for generating their global collaborative network with VOSViewer, as shown in Fig. 10.

It is unsurprising to see that European countries occupy a significant position in collaborating with high-burden countries, especially the United Kingdom, which has demonstrated a strong partnership with Malawi. The United States is also a key partner to these countries as well as with Zimbabwe. Another quite remarkable point is the cohesive collaboration between African countries. South Africa, as a key academic contributor in Africa, plays a vital role in the collaborative networks of high-burden countries. Swaziland and Lesotho have the strongest collaboration. This is perhaps to be expected since the two countries are highly similar in geographical location, language, and culture. Compared with other continents, collaborations between Asian countries and Africa are relatively weak. Notably, China’s collaborations with the high-burden countries do not even match Mongolia, Thailand or several other countries in the region.

From the perspective of aggregate level of HDI groups, the strongest collaborators over the past two decades with the five greatest-burden countries have been those in the very-high group, followed by those in the low group, who also suffer from great IPDs burden. Together with the results from the “By country” section, this analysis further reveals that the most research is produced by African countries focused on their local burden. However, enriching this insight is the finding that some of this effort involves relatively strong collaborations with highly developed regions. This implication confirms Confraria and Wang’s [30] conclusion that there is no clear trade-off for African countries between participating in global research networks and producing medical research that is aligned with local health needs.

## Conclusion and discussion

### Summary

IPDs are one of the leading causes of death and disability and are responsible for a significant burden on public health and social progress. As an essential means by which humans can fight against various diseases, how academics react to the severe health needs caused by IPDs is a vital question to be explored. Thus, this international review of what, where and how research has been done and how it matches with societal needs is an insightful source of knowledge for ensuring an efficient global research effort. In this paper, we investigated the response patterns of academic research to IPDs based on the dynamic relationship between disease burden and research effort on IPDs, the scientific efforts contributed by countries with different development levels, and the variation trends in international collaborations.

In terms of the dynamic relationship between disease burden and research effort on IPDs, the percentage of DALYs is still greater than that of publications in general, indicating the need for a continuous scientific focus on IPDs. More importantly, our findings reveal a substantially diminishing gap and a positive correlation between the level of research efforts and disease burden globally. Given that the burden of several high-burden IPDs has decreased considerably with effective treatments, we argue that related research could focus more on social, economic and environmental issues with regard to IPDs in underdeveloped regions, such as the accessibility of healthcare. As for specific diseases, we observed that diseases (hepatitis) with stable burden have gained greater attention as the overall burden of IPDs has decreased, which to some degree reflects the sensitivity and responsiveness of research efforts with respect to disease burden. This is also demonstrated by the positive correlation between the level of research efforts and burden globally.

In terms of the regional distribution, IPDs burden is mainly concentrated in the less developed regions in Africa, while related academic research is mainly from developed continents. Indeed, those highly developed countries account for nearly two thirds of all research outputs in terms of absolute number of publications. However, according to the SI, the underdeveloped area (low-level group) is not only the region with the largest IPDs burden, but also the region putting forth the most effort to research IPDs. Such a result again illustrates the alignment of disease burden and research effort from the perspective of geographical distribution. Furthermore, while the worldwide IPDs burden has decreased dramatically, the NSI value of IPDs burden in low-level countries is growing, highlighting the need for continued attention to disease burden in undeveloped regions. Simultaneously, there is a clear increase in research efforts from regions with the heaviest disease burden, despite their limited research capacity. In fact, apart from the constantly increasing number of publications produced by the least developed regions, the distribution of their research efforts on the 11 specific conditions also correlates with the distribution of burden in those regions, in which parasitic and vector diseases is the one with the highest burden and receives the most attention. In particular, Uganda stands out as a compelling example of diligence to its local health needs and achieving remarkable effects on alleviating their burden.

In terms of academic collaboration, our analysis shows that both the number of collaborative publications and the collaboration strength has increased remarkably in the past two decades across the board. Collaborations are most intense between the very-high and low groups. In particular, the United States and United Kingdom play a prominent role in collaborating with high-burden countries. In fact, a cohesive collaboration within African countries can also be observed from this study. As for the dynamic trend, the strength of African countries’ collaboration with the very-high and medium groups is expanding, implying improvements in research and collaborative efforts of low-level groups with different regions. Given their own limited resources, low-level countries not only seek collaboration with highly developed countries to jointly handle their local health problems, but also unite areas with similar health needs. Moreover, among all the collaboration pairs, the high–low collaborations generate the fewest publications and are the weakest. Additionally, China, a high (medium)-level country, has a relatively weak collaboration with high-burden countries despite its place as the third highest research contributor. Such rapidly developing countries could also seek increased engagement and pursue a more active role in research on global needs.

We also noted that there is no uniform standard or requirement for academic contribution for each country across the different development levels. The uneven distribution of research effort by the different nations once again raises the issue of whether research effort should be balanced between global and local needs or whether a local focus is optimal. Our analysis reveals a tendency for all countries of the world, no matter their development level, to concentrate on their own issues. This is a finding that is consistent with previous studies [26, 38] but one that, no doubt, will stoke further lively debate. Indeed, local and global health needs are both relative concepts. In this study, IPDs is regarded as a local health concern for underdeveloped regions and a global health issue confronting highly developed regions. Focusing on local needs might be the optimal option for resource-constrained regions. Hence, enhancing scientific capability in low-level regions is the primary strategy for addressing their local burden. In addition to focusing on local needs, conducting research that extends its impact beyond national borders is a consideration for regions with scientific capacity.

### Limitations and future research

This study has several limitations that need to be addressed. First, the only measure of research effort was publication counts from WoS. Drawing only from WoS might mean that the corpus did not include some relevant publications indexed in other scholarly databases. Moreover, while publication counts are an objective measure, they do not represent all types of research efforts. The same is true for the burden of disease. A wide range of indicators beyond DALYs have been developed to monitor and manage health initiatives [75, 76]. The use of these indicators may produce different results. Hence, these empirical results need to be interpreted and applied to policy-making with caution due to their different computing logics, limitations and applicable scenarios. In addition, several diseases with ambiguous titles, such as other infectious diseases in IPDs and other neoplasms, are excluded owing to the need for accurate information for retrieving publication data, which is also one of the limitations of this study.

Second, it is controversial whether research effort and academic resources should be allocated relative to the health needs or more directly to the burden of disease [77]. A range of factors influence what types of research receive priority. Additionally, alleviating the burden of disease and addressing health needs not only involves an endless research effort; it also relies on the appropriateness of practices, access to prevention and treatment facilities, medical quality controls and the financing of the healthcare system. Hence, we acknowledge that measuring the association between research effort and disease burden is not straightforward; multiple societal factors affect both sides. In practice, it might be impossible to quantify the optimal relationship between a disease and its corresponding R&D efforts for any disease or group of diseases. Nevertheless, gaining a thorough and systematic view of the dynamic relationship between research efforts and health needs is the first step towards understanding the complex, multidimensional and integrated interaction mechanisms between science and society. Moreover, it is undeniable that this is the most transparent and fair way to quantitatively explore the dynamic relationship between health needs and research effort. Such insights are useful when setting priorities for R&D investment [48] and for providing explicit empirical evidence on these issues to policy-makers and the public [43].

For future research, multidimensional indices should be expanded to provide a more comprehensive understanding of the relationship between research efforts and societal demands in medical fields. Diverse societal factors relevant to health needs could be included to more comprehensively investigate the reasons behind the relationship between academic research and social needs. And, as always, the balance of research effort between global and local needs for different countries warrants further exploration.

## Data Availability

The raw bibliometric data were collected from the Clarivate Analytics WoS, which is a commercial database requiring a license to access. The raw data on disease burden and the HDI were downloaded from the WHO and UNDP websites, respectively. Web addresses for obtaining disease burden data and HDI are provided in the “Data and methodology” section in the article.

## Appendices

### A1. The search strategy on publications

The publications on the 11 specific conditions in IPDs and other categories are collected according to search terms built by Confraria and Wang [30]. These terms were derived from the diseases listed in the Global Health Estimates (GHE) cause categories established according to International Classification of Diseases, Tenth Revision (ICD-10) by WHO [46] (Fig. 11).

As mentioned in the “Health needs” section, the GHE cause category has a hierarchical structure of four levels containing three broad groups. As for the publication data retrieval, the search terms of five subgroups in group I and 13 subgroups in group II are included, in which the search strategies for all 11 conditions (level 3) in IPDs are also provided in a more fine-grained approach by Confraria and Wang [30]. Moreover, it should be noted that the terms “all causes/diseases” used in the text refer to the sum of the five subgroups in group I and 13 subgroups in group II. Correspondingly, “burden (publications) of all causes/diseases” also indicates the sum of the disease burden (publications) in the above 18 subgroups. The other subgroups (e.g. “other neoplasms”) in group II are excluded due to ambiguity. Further, because group III (injuries category) mainly refers to transportation and intentional/unintentional injuries rather than specific diseases, we did not include it in this study.

The search terms on each of the 11 specific conditions in IPDs and diseases of other subgroups are applied in the “TI” (title) and “AK” (author keywords) fields in advanced research in WoS to obtain publications (articles and reviews) on IPDs and all the other diseases. Table 6 provides details on the search terms.

**Table 6:** Search terms for each disease [30]

(a) Eleven conditions of IPDs		
Code	Diseases	Search terms
1	Tuberculosis	"tuberculosis" OR "tubercolosis" OR "tubercle bacillus" OR "tuberculin" OR "tb infection" OR "pulmonary tb" OR "extrapulmonarytb"
2	STDs excluding HIV	"Syphilis" OR "Chlamydia" OR "Gonorrhoea" OR "Trichomoniasis" OR "Genital herpes"
3	HIV/AIDS	("hiv/aids" OR "human immunodeficiency virus" OR "human immuno-deficiency virus" OR "acquired immunodeficiency syndrome" OR "acquired immuno-deficiency syndrome" OR "hiv infection") NOT (feline OR simian)
4	Diarrheal diseases	"Diarrhoeal" OR "diarrhoea" OR "E. coli" OR "E. Coli" OR "V. cholerae" OR "shigellosis" OR "shigella" OR "Giardia" OR "cryptosporidium" OR "rotavirus"
5	Childhood-cluster diseases	"Whooping cough" OR "pertussis" OR "Diphtheria" OR "diphtheriae" OR "Measles" OR "rubeola" OR "neonatal tetanus" OR "tetanus neonatal" OR "mumps virus" OR "Poliomyelitis"
6	Meningitis	"Meningitis" OR "meningitidis" OR "neisseria pneumoniae" OR "cryptococc*" OR "meningococcus"
7	Encephalitis	"Encephalitis"
8	Hepatitis	"Hepatitis"
9	Parasitic and vector diseases	(("Malaria" OR "plasmodium" OR "anopheles" OR "black water fever") NOT "physarum") OR "Human african trypanosomiasis" OR "sleeping sickness" OR "trypanosom human" OR "Chagas disease" OR "American Trypanosomiasis" OR "Trypanosoma cruzi" OR "Trypanosoma brucei" OR "Schistosomiasis" OR "bilharzia" OR "Schistosoma mansoni" OR "Schistosoma haematobium" OR "Schistosoma intercalatum" OR "Schistosoma japonicum" OR "Schistosoma mekongi" OR "Leishmaniasis" OR "Leishmania" OR "phlebotomine" OR "psychodidae" OR "kalaazar" OR "kala-azar" OR "kala azar" OR "sand fly" OR "sandflies" OR "sand flies" OR "filariasis" OR "elephantiasis" OR "wuchereria" OR "brugia malayi" OR "Onchocerciasis" OR "Onchoceriasis" OR "river blindness" OR "onchocerca volvulus" OR "Cysticercosis" OR "taeniasis" OR "Taenia solium" OR "Echinococcosis" OR "hydatid disease" OR "echinococcus" OR "dengue" OR "aedes aegypti" OR "aedes albopictus" OR "Trachoma" OR "chlamydia trachomatis" OR "Yellow fever" OR "Rabies" OR "zika virus" OR "Flavivirus" OR "chikungunya" OR "Lassa fever" OR "Ebola" OR "Haemorrhagic Fever" OR "typhoid" OR "loiasis" OR "cestodes"
10	Intestinal nematode infections	(("fasciolosis" OR "fascioliasis" OR "distomatosis" OR "fasciola hepatica" OR "fasciola gigantica" OR "distomatosis") NOT "cattle") OR "dracunculiasis disease" OR "guinea-worm disease" OR "guinea worm disease" OR "dracunculus medinensis" OR "salmonella" OR "paratyphoid fever" OR "ancylostomiasis" OR "strongyloidiasis" OR "Ascariasis" OR "Trichuriasis" OR "Hookworm*" OR "heminth*" OR "hook-worm*" OR "hook worm*" OR "ascaris lumbricides" OR "trichuris trichiura" OR "geohelminth*" OR "necatoramericanus" OR "necator americanus" OR "necatoriasis" OR "ancylostoma duodenale" OR "ancylostoma-duodenale" OR "clonorchiasis" OR "opisthorchiasis" OR "paragonimiasis"
11	Leprosy	"Leprosy" OR "hansen disease" OR "mycobacterium leprae"

(b) Other diseases in GHE cause category	
Diseases	Search terms
Respiratory infections & diseases	"respiratory infectio*" OR "Asbestosis" OR "rheumatic fever" OR "Haemophilus Influenzae" OR "lung absces" OR "bronchitis" OR "Streptococcus pneumoniae" OR "pneumonia" OR "Moraxella catarrhalis" OR "Klebsiella pneumonia" OR "tonsillitis" OR "rhinitis" OR "sinus infection" OR "sinusitis" OR "rhinosinusitis" OR "rhinopharyngitis" OR "nasopharyngitis" OR "pharynx inflammation" OR "hypopharynx inflammation" OR "uvula inflammation" OR "tonsils inflammation" OR "pharyngitis" OR "epiglottitis" OR "laryngitis" OR "laryngotracheitis" OR "tracheitis" OR "Otitis media" OR "Respiratory diseas*" OR "Chronic obstructive pulmonary disease" OR "Asthma" OR "emphysema" OR "Laryngotracheitis" OR "Epiglottitis" OR "Bacterial tracheitis"
Maternal conditions	"maternal death" OR "maternal mortality" OR "pregnancy infection*" OR "abortion care" OR "unsafe abortion" OR "childbirth severe bleeding" OR "childbirth infection*" OR "Placental abruptio*" OR "placenta praevia"
Neonatal conditions	"Preterm birth" OR "Birth asphyxia" OR "birth trauma" OR "Neonatal sepsis" OR "neonatal infection*" OR "Gastroschisis" OR "Jaundice" OR "Necrotizing enterocolitis" OR "Persistent pulmonary hypertension of the newborn" OR "Intrauterine growth restriction" OR "Bronchopulmonary dysplasia" OR "infant apnoea" OR "infant respiratory distress syndrome" OR "asphyxia at birth" OR "anaemia in neonates" OR "neonatal alloimmune thrombocytopenia" OR "bronchopulmonary dysplasia" OR "cardiac failure in neonates" OR "hyaline membrane disease" OR "hypocalcaemia in neonates" OR "hypoglycaemia of the newborn" OR "hyponatraemia in neonates" OR "hypothermia in neonates" OR "intestinal obstruction in neonates" OR "pulmonary interstitial emphysema”
Nutritional deficiencies	"Nutritional deficienc*" OR "Protein energy malnutrition" OR "Protein-energy malnutrition" OR "Iodine deficiency" OR "Irondeficiency anaemia" OR "Nutritional deficienc*" OR "Thiamine deficiency" OR "vitamin B-1 deficiency" OR "Niacin deficiency" OR "vitamin B-3 deficiency" OR "vitamin B-9 deficiency" OR "Folate deficiency" OR "Cobalamin deficiency" OR "vitamin B-12 deficiency" OR "Vitamin D deficiency" OR "Calcium deficiency" OR “marasmus” OR “Kwashiarkor” OR “Marasmic-kwashiorkor” OR “nutritional oedema” OR “severe acute malnutrition” OR "moderate acute malnutrition” OR "Vitamin A deficiency"
Malignant neoplasms	"malignant neoplasm*" OR "Mouth cancer" OR "oropharynx cancer" OR "Lip cavity" OR "oral cavity" OR "Nasopharynx" OR "Oesophagus cancer" OR "Stomach cancer" OR "Colon cancer" OR "rectum cancer" OR "Liver cancer" OR "Pancreas cancer" OR "Trachea cancer" OR "bronchus cancer" OR "lung cancer" OR "Melanoma" OR "skin cancer" OR "Breast cancer" OR "Cervix uteri cancer" OR "Corpus uteri cancer" OR "Ovary cancer" OR "Prostate cancer" OR "Testicular cancer" OR "Kidney cancer" OR "Bladder cancer" OR "Brain cancer" OR "nervous system cancer" OR "Gallbladder cancer" OR "biliary tract cancer" OR "Larynx cancer" OR "Thyroid cancer" OR "Mesothelioma" OR "Lymphoma*" OR "multiple myeloma" OR "Leukaemia"
Diabetes mellitus	diabete*
Endocrine, blood, immune disorders	"Endocrine disorder*" OR "blood disorder*" OR "immune disorder*" OR "Glucocorticoid deficiency" OR "Glucose intolerance" OR "goitre" OR "Hyperparathyroidism" OR "Hyperthyroidism" OR "Hypoglycemia" OR "Hypoparathyroidism" OR "Hypothyroidism" OR "Mineralocorticoid deficiency" OR "Pseudohypoparathyroidism" OR "Thyroid cyst" OR "Thyroid nodule" OR "Thyroiditis" OR "Acidosis" OR "Alkalosis" OR "Amyloidosis" OR "Thalassaemias" OR "Sickle cell disorder" OR "trait disorder" OR "haemoglobinopathies" OR "haemolytic anaemia" OR "Cystic fibrosis" OR "Dysmetabolic syndrome" OR "Hemochromatosis" OR "Hyperbilirubinemia" OR "Hypercalcemia" OR "hypercholesterolaemia" OR "Hyperkalemia" OR "hyperlipidaemia" OR "Hypernatremia" OR "Hypertriglyceridemia" OR "Hypocalcemia" OR "Hypokalemia" OR "Hyponatremia" OR "Hypovolemia" OR "Magnesium disorder*" OR "Obesity hypoventilation syndrome" OR "Porphyria" OR "Renal osteodystrophy" OR "anaemia" OR "Coagulation defects" OR "Eosinophilia" OR "haemophilia" OR "Hypercoagulable state" OR "Idiopathic thrombocytopenic purpura" OR "Leukocytopenia" OR "Leukocytosis" OR "Lymphadenitis" OR "Neutropenia" OR "Polycythemia vera" OR "Sickle cell" OR "Thrombocytopenia"
Mental and substance use disorders	"mental disorder*" OR "substance disorder*" OR "behavioural disorder*" OR "Agoraphobia" OR "Anorexia nervosa" OR "Antisocial personality disorder" OR "Anxiety state" OR "Attention deficit" OR "hyperactivity" OR "Bipolar disorder" OR "Borderline personality disorder" OR "Bruxism" OR "Bulimia nervosa" OR "Conduct disorder" OR "Conversion disorder" OR "Delirium tremens" OR "Dementia" OR "Depression disorder" OR "Depressive disorder" OR "Depressive psychosis" OR "Dyspareunia" OR "Encopresis" OR "Enuresis" OR "Explosive personality disorder" OR "Fluency disorder" OR "Generalized anxiety disorder" OR "Hysteria disorder" OR "Hysterical psychosis" OR "Insomnia" OR "sleep disorder" OR "Intellectual disabilit*" OR "Neurosis" OR "Neurotic depression" OR "Obsessive–compulsive disorder" OR "Panic disorder" OR "Paranoid reaction" OR "Personality disorder" OR "Post-traumatic stress disorder" OR "Premature ejaculation" OR "Psychosis" OR "Schizoaffective" OR "Schizophrenia" OR "Sleep disorder" OR "Somatization disorder" OR "Somnambulism" OR "Suicidal ideation" OR "Alcohol abuse" OR "Alcoholism" OR "Amphetamine dependence" OR "Cannabis abuse" OR "Cannabis dependence" OR "Cocaine abuse" OR "Cocaine dependence" OR "Drug abuse" OR "Drug withdrawal" OR "Drug-induced paranoia" OR "Opioid abuse" OR "Opioid dependence" OR "Tobacco abuse" OR "dysthymia" OR "opioid disorder" OR "cocaine disorder" OR "amphetamine disorder" OR "cannabis disorder" OR "panic attack" OR "Social anxiety disorder" OR "separation anxiety disorder" OR "selective mutism" OR "eating disorder" OR "Anorexia Nervosa" OR "Bulimia Nervosa" OR "Binge Eating Disorder" OR "Muscle dysmorphia" OR "autism" OR "asperger syndrome" OR "autistic" OR "Attention deficit" OR "hyperactiv* syndrome" OR "Conduct disorder" OR "Idiopathic intellectual disability" OR "mental retardation"
Neurological conditions	"Bell's palsy" OR "Blepharospasm" OR "Carpal tunnel" OR "Cerebral aneurysm" OR "Cerebral artery occlusion" OR "Cerebral oedema" OR "Cerebral palsy" OR "Cognitive impairment" OR "Encephalopathy" OR "Epilepsy" OR "Guillain-Barré" OR "Hemiplegia" OR "Hydrocephalus" OR "Migraine" OR "Morton's neuroma" OR "Multiple sclerosis" OR "Myasthenia gravis" OR "Narcolepsy" OR "Neuralgia" OR "Neuropathy" OR "Parkinsonism" OR "Phantom limb" OR "Post-concussion syndrome" OR "Postherpetic neuralgia" OR "Pseudotumor cerebri" OR "Reflex sympathetic" OR "Restless legs syndrome" OR "Reye's syndrome" OR "Sciatica" OR "Subarachnoid haemorrhage" OR "Subdural haemorrhage" OR "Thoracic outlet syndrome" OR "Tic disorder" OR "Tourette's disorder" OR "Trigeminal neuralgia" OR "Alzheimer*" OR "dementia" OR "chronic neurodegenerative" OR "Neurodegeneration" OR "Parkinson disease" OR "parkinsonian syndrome" OR "epileptic" OR "motor neuron disease" OR "huntington's disease"
Sense organ diseases	"otitic barotrauma" OR "Cerumen impaction" OR "Eustachian tube dysfunction" OR "Hearing loss" OR "viral Labyrinthitis" OR "Ménière's disease" OR "Nystagmus" OR "Otalgia" OR "Otitis externa" OR "Otitis media" OR "Presbycusis" OR "Tinnitus" OR "Vertigo" OR "Anisocoria" OR "Blepharitis" OR "eye cataract*" OR "Chalazion" OR "Conjunctivitis" OR "Corneal abrasion" OR "Corneal oedema" OR "Corneal ulcer" OR "Diplopia" OR "Dry eye syndrome" OR "Esotropia" OR "Glaucoma" OR "Hyphema" OR "Iritis" OR "cyclitis" OR "Lid lag" OR "Macular degeneration" OR "Papilledema" OR "Pterygium" OR "Retinal detachment" OR "Retinopathy" OR "Scotoma" OR "hordeolum" OR "Subconjunctival haemorrhage" OR "Visual disturbance" OR "Visual field defect" OR "Visual loss" OR "Uncorrected refractive errors" OR "sense organ disorder*" OR "cholesteatoma"
Cardiovascular diseases	"Cardiovascular diseas*" OR "Atrial fibrillation" OR "Atrial flutter" OR "Atrioventricular block" OR "Bundle branch block" OR "Long QT syndrome" OR "Sick sinus syndrome" OR "Sinoatrial heart block" OR "Sinus bradycardia" OR "paroxysmal tachycardia" OR "Angina pectoris" OR "artery bypass graft" OR "autologous vein bypass graft" OR "native coronary artery" OR "Cardiac arrest" OR "Cardiac contusion" OR "Cardiomyopathy" OR "Chronic ischaemic heart disease" OR "Endocarditis" OR "Heart failure" OR "Heart valve" OR "Kawasaki disease" OR "Myocarditis" OR "Pericarditis" OR "Prinzmetal angina" OR "Pulmonary heart disease" OR "Rheumatic heart disease" OR "Aortic aneurysm" OR "Aortic dissection" OR "Carotid sinus syndrome" OR "Deep vein thrombosis" OR "oesophageal varices" OR "heart hypertension" OR "Hypertensive heart" OR "Hypotension" OR "Intermittent claudication" OR "Peripheral vascular disease" OR "Phlebitis" OR "Polyarteritis nodosa" OR "Postmastectomy lymphedema" OR "Raynaud's syndrome" OR "Thrombophlebitis" OR "Transient ischaemic attack" OR "Varicose veins" OR "Venous embolism" OR "Venous insufficiency" OR "Wegener's granulomatosis" OR "Ischaemic heart disease" OR "Ischaemic stroke" OR "Haemorrhagic stroke" OR "myocardial infarction"
Digestive diseases	"Digestive diseas*" OR "Achalasia" OR "cardiospasm" OR "Anal spasm" OR "Angiodysplasia" OR "Aphthous ulcer" OR "Appendicitis" OR "Barrett's esophagitis" OR "Cholangitis" OR "Cholecystitis" OR "Cholelithiasis" OR "Cirrhosis" OR "Crohn's disease" OR "Diverticulitis of colon" OR "Duodenal ulcer" OR "Dyspepsia" OR "Edentulism" OR "oesophageal stricture" OR "oesophageal stenosis" OR "Esophagitis" OR "Fatty liver" OR "Gallbladder disease" OR "Gastric ulcer" OR "Gastritis" OR "Gastroenteritis" OR "Gastroesophageal reflux" OR "Gastroparesis" OR "Glossitis" OR "Hemorrhoids" OR "Impaction of intestine" OR "colostomy" OR "enterostomy" OR "Irritable bowel syndrome" OR "ischaemic bowel disease" OR "Leukoplakia" OR "Liver disease" OR "Mechanical complication of ostomy" OR "Pancreatitis" OR "Parotitis" OR "Peptic ulcer" OR "Periodontitis" OR "Ulcerative colitis" OR "duodenitis" OR "Paralytic ileus" OR "intestinal obstruction" OR "Inflammatory bowel disease"
Genitourinary diseases	"Genitourinary" OR "Breast lump" OR "Fibroadenosis" OR "Fibrocystic disease" OR "Galactorrhea" OR "gynaecomastia" OR "Mastitis" OR "Mastodynia" OR "amenorrhoea" OR "Menopausa*" OR "Metrorrhagia" OR "Mittelschmerz" OR "Premenstrual tension syndrome" OR "postmenopausal atrophic" OR "vulvo atrophy" OR "vaginal atrophy" OR "Bartholin abscess" OR "Bartholin cyst" OR "Cervical polyp" OR "Cervicitis" OR "Corpus luteum cyst" OR "Cyst of ovary" OR "Cystocele" AND "midline" OR "Dyspareunia" OR "Endometrial hyperplasia" OR "Endometriosis" OR "Fibroid uterus" OR "leiomyoma" OR "Leukorrhea" OR "Ovarian failure" OR "Pelvic inflammatory disease" OR "uterine prolapse" OR "Rectocele" OR "Urethrocele" OR "Uterus hypertrophy" OR "Vaginismus" OR "Vaginitis" OR "vulvitis" OR "Vulvodynia" OR "Atrophy of testis" OR "Balanitis" OR "BPH/LUTS" OR "Hematospermia" OR "Hydrocele" OR "Orchitis" OR "epididymitis" OR "Phimosis" OR "Priapism" OR "Prostatitis" OR "Spermatocele" OR "Testicular hypofunction" OR "Torsion of testis" OR "nongonococcal Urethritis" OR "Varicocele" OR "Atony of bladder" OR "Bladder hypertonicity" OR "Bladder neck obstruction" OR "kidney Calculus" OR "ureter Calculus" OR "urinary Calculus" OR "Cystitis" OR "Glomerulonephritis" OR "haematuria" OR "Hydronephrosis" OR "Kidney disease" OR "Nephrotic syndrome" OR "Proteinuria" OR "Pyelonephritis" OR "Renal failure" OR "urethral stricture" OR "Urethral syndrome" OR "Urinary obstruction" OR "Urinary tract infection" OR "Vesicoureteral" OR "prostatic hyperplasia" OR "Urolithiasis" OR "gynaecologic* disease" OR "infertility"
Skin diseases	"skin diseas*" OR "Acne" OR "Actinic keratosis" OR "Alopecia" OR "Cellulitis" OR "Contact dermatitis" OR "Cradle cap" OR "Dermatitis" OR "Dermatophytosis" OR "Diaper rash" OR "Eczema" OR "Erythema multiforme" OR "Erythema nodosum" OR "Hidradenitis suppurativa" OR "Hirsutism" OR "Impetigo" OR "Ingrown nail" OR "Keloid scar" OR "Lichen planus" OR "Lymphadenitis" OR "Onychomycosis" OR "Paronychia" OR "Pityriasis rosea" OR "Pressure ulcer" OR "Pruritus" OR "Psoriasis" OR "Sebaceous cyst" OR "seborrhoeic dermatitis" OR "seborrhoeic keratosis" OR "Solar radiation dermatitis" OR "Stevens-Johnson syndrome" OR "Tinea cruris" OR "Tinea pedis" OR "Tinea versicolor" OR "Urticaria" OR "Vitiligo"
Musculoskeletal diseases	"Musculoskeletal disease" OR "Musculoskeletal disorder" OR “Musculoskeletal pain” OR "Arthropathy" OR "Dermatomyositis" OR "Eosinophilia myalgia syndrome"OR "Fibromyalgia"OR "Myositis ossificans" OR "Osteoarthrosis" OR "Osteochondritis" OR "Osteomyelitis" OR "Osteoporosis" OR "Polymyalgia rheumatica" OR "Polymyositis" OR "Rhabdomyolysis" OR "Sjögren's disease" OR "Synovitis" OR "tenosynovitis" OR "Systemic lupus erythematosus" OR "Temporomandibular arthralgia" OR "Aseptic necrosis"OR "Baker's cyst" OR "Bunion" OR "Calcaneal spur" OR "Chondromalacia of patella" OR "knee* derangement" OR "Hallux rigidus" OR "Hallux valgus" OR "Hammer toe" OR "Iliotibial band syndrome" OR "Knee effusion" OR "Metatarsalgia" OR "Pes anserinus tendinitis" OR "Plantar fasciitis" OR "Prepatellar bursitis" OR "Tendinitis" OR "Tenosynovitis" OR "Ankylosing spondylitis" OR "Cervical spondylosis" OR "Coccygodynia" OR "Costochondritis" OR "Degenerative disc disease" OR "Diastasis recti" OR "Kyphosis" OR "Lumbosacral spondylosis" OR "Postlaminectomy syndrome" OR "Sacroiliitis" OR "Scoliosis" OR "Somatic dysfunction"OR "Spinal stenosis" OR "Spondylolisthesis" OR "Thoracic spondylosis" OR "Torticollis" OR "Adhesive capsulitis" OR "Bicipital tenosynovitis" OR "Boutonniere deformity" OR "de Quervain's disease" OR "Dupuytren's contracture" OR "Lateral epicondylitis" OR "Mallet finger" OR "Medial epicondylitis" OR "Olecranon bursitis" OR "Swan-neck deformity" OR "Tenosynovitis" OR "Rheumatoid arthritis" OR "Osteoarthritis" OR "Gout" OR "Back pain" OR "neck pain" OR "Osteomyelitis"
Congenital anomalies	"Arteriovenous malformation" OR "Atrial septal defect" OR "Hirschsprung's disease" OR "Hydrocephalus" OR "Hypospadias" OR "Imperforate anus" OR "Imperforate hymen"OR "Limb anomaly" OR "Marfan syndrome" OR "Meckel's diverticulum" OR "Microcephalus" OR "Osteogenesis imperfecta" OR "Pectus excavatum" OR "Pyloric stenosis" OR "Spina bifida" OR "Talipes equinovarus" OR "Tongue tie" OR "Congenital Muscular Torticollis" OR "Congenital Torticollis" OR "Undescended testis" OR "Ventricular septal defect" OR "Neural tube defects" OR "Cleft lip" OR "cleft palate" OR "Down syndrome" OR "trisomy" OR "Down's syndrome" OR "Congenital heart anomal*" OR "Congenital anomal*"
Oral conditions	"Oral disorder" OR "oral disease" OR "mouth disease" OR "oral cancer" OR "Gingivitis" OR "Thrush" OR "Mouth Ulcer" OR "dental carie*" OR "periodontal disease" OR "edentulism" OR "tooth decay"

### A2. The normalized publication specialization index ($\mathrm{NSI}\_\mathrm{Pub}$) by human development level (all diseases)

**Table:**

	Infectious and parasitic diseases	Respiratory infections/diseases	Maternal conditions	Neonatal conditions	Nutritional deficiencies		Malignant neoplasms		Diabetes mellitus		Endocrine, blood, immune disorders		Mental disorders and substance abuse		Neurological conditions		Sense organ diseases		Cardiovascular diseases	Digestive diseases	Genitourinary diseases	Skin diseases	Musculoskeletal diseases	Congenital anomalies	Oral conditions
2000–2004																									
Low	0.52	−0.29	0.76	−0.21	0.56		−0.50		−0.11		−0.04		−0.56		−0.42		−0.15		−0.60	−0.25	−0.01	−0.04	−0.54	−0.31	0.34
Med	0.31	−0.08	0.22	0.08	0.34		−0.24		−0.06		−0.02		−0.25		−0.15		−0.04		−0.21	−0.02	0.07	−0.08	−0.08	−0.01	0.25
High	0.11	0.00	0.10	−0.19	0.05		−0.08		0.12		0.04		−0.21		−0.02		−0.15		−0.02	0.01	0.09	0.01	−0.03	−0.07	0.00
Very high	−0.09	0.01	−0.15	0.01	−0.10		0.03		0.00		0.00		0.04		0.02		0.01		0.03	0.01	−0.01	0.01	0.02	0.01	−0.05
2005–2009																									
Low	0.51	−0.20	0.70	0.03		0.50		−0.41		−0.03		0.02		−0.51		−0.32		−0.09	−0.50	−0.16	0.01	−0.06	−0.42	−0.13	0.24
Med	0.21	−0.02	0.05	−0.03		0.21		−0.09		−0.02		−0.06		−0.25		−0.14		0.05	−0.12	0.06	0.10	0.09	0.02	0.05	0.08
High	0.27	0.01	0.18	−0.07		0.23		−0.25		0.06		0.05		−0.14		−0.07		−0.09	−0.10	−0.03	0.04	−0.05	−0.12	−0.06	0.36
Very high	−0.11	0.01	−0.15	0.01		−0.10		0.04		0.00		0.00		0.04		0.03		0.00	0.03	0.00	−0.02	0.00	0.02	0.00	−0.07
2010–2014																									
Low	0.58	−0.21	0.81	0.09		0.43		−0.58		−0.19		0.00		−0.60		−0.56		−0.20	−0.54	−0.40	−0.18	−0.37	−0.66	−0.24	0.08
Med	0.20	−0.09	0.05	−0.14		0.02		0.13		0.03		−0.17		−0.36		−0.12		−0.01	−0.18	0.03	0.00	−0.11	−0.04	−0.09	0.23
High	0.20	−0.02	0.05	−0.07		0.17		−0.22		0.03		0.05		−0.17		−0.10		0.00	−0.10	0.01	0.11	0.12	0.00	−0.02	0.28
Very high	−0.12	0.02	−0.13	0.03		−0.05		0.01		−0.01		0.02		0.07		0.04		0.00	0.04	0.00	−0.01	0.00	0.02	0.02	−0.12
2015–2019																									
Low	0.52	−0.07	0.75	0.13		0.59		−0.52		0.11		0.10		−0.45		−0.38		−0.19	−0.35	−0.15	−0.02	−0.26	−0.41	−0.19	0.11
Med	0.33	−0.01	0.42	0.07		0.32		−0.19		0.09		0.06		−0.40		−0.17		0.11	−0.32	0.01	0.08	0.11	−0.15	0.01	0.33
High	0.10	−0.03	−0.18	−0.11		−0.03		0.11		0.04		−0.16		−0.23		−0.08		−0.07	−0.09	0.02	0.01	−0.10	0.04	−0.14	0.16
Very high	−0.13	0.01	−0.13	0.02		−0.08		−0.01		−0.03		0.03		0.08		0.04		0.01	0.05	0.00	−0.01	0.02	0.01	0.04	−0.12

## Abbreviations

AIDS: Acquired immunodeficiency syndrome
DALYs: Disability-adjusted life years
GHE: Global Health Estimates
HBV: Hepatitis B virus
HDI: Human Development Index
HIV: Human immunodeficiency virus
ICD-10: International Classification of Diseases, Tenth Revision
IPDs: Infectious and parasitic diseases
NSI: Normalized specialization index
PHEIC: Public health emergency of international concern
R&D: Research and development
SI: Specialization index
UN: United Nations
UNDP: United Nations Development Programme
WHA: World Health Assembly
WoS: Web of Science

## Footnotes

1 The third mission refers to contributions of universities to the society.

2 Please see Appendix A1 for a more detailed explanation.

3 https://www.vosviewer.com/.

4 HIV stands for human immunodeficiency virus, which targets the immune system and weakens people’s defence against many infections. The most advanced stage of HIV infection is acquired immunodeficiency syndrome, or AIDS, which is characterized by the development of certain cancers, infections or other severe long-term clinical manifestations.

5 The number in parentheses after the name of a disease corresponds to the number on the horizontal axis of Fig. 6 or 7.

6 Antarctica excluded.

7 A publication generated within the same HDI group could refer to the output of a single country within this group or a collaboration of multiple countries within this group.
